# Cytotoxic and *N*-Acetyltransferase Inhibitory Meroterpenoids from *Ganoderma cochlear*

**DOI:** 10.3390/molecules23071797

**Published:** 2018-07-20

**Authors:** Li-Zhi Cheng, Fu-Ying Qin, Xiao-Chi Ma, Shu-Mei Wang, Yong-Ming Yan, Yong-Xian Cheng

**Affiliations:** 1 School of Traditional Chinese Medicine, Guangdong Pharmaceutical University, Guangzhou 510006, China; 13424039397@163.com (L.-Z.C.); shmwang@sina.com (S.-M.W.); 2 Guangdong Key Laboratory for Genome Stability & Disease Prevention, School of Pharmaceutical Sciences, Shenzhen University Health Science Center, Shenzhen 518060, China; qinfuying@mail.kib.ac.cn (F.-Y.Q.); yanym@szu.edu.cn (Y.-M.Y.); 3 State Key Laboratory of Phytochemistry and Plant Resources in West China, Kunming Institute of Botany, Chinese Academy of Sciences, Kunming 650201, China; 4 School of Pharmaceutical Sciences, Dalian Medical University, Dalian 116044, China; maxc1978@sohu.com

**Keywords:** *Ganoderma cochlear*, meroterpenoids, cytotoxic activity, *N*-acetyltransferase

## Abstract

Seven compounds, including two pairs of new meroterpenoids, (+)- and (−)-gancochlearol C (**1**), (+)- and (−)-cochlearoid Q (**3**), and a new meroterpenoid gancochlearol D (**2**), together with four known meroterpenoids were isolated from the aqueous EtOH extract of the fruiting bodies of *Ganoderma cochlear.* Their structures were determined by spectroscopic data. The isolated compounds were evaluated for their cytotoxic activity against three human lung cancer cells (H1975, PC9, A549) and *N*-acetyltransferase inhibitory property. The results show that (+)-gancochlearol C could inhibit *N*-acetyltransferase with an IC_50_ value of 5.29 μM. In addition, ganomycin F was found to show moderate activity against the H1975 human lung cancer cell line, with an IC_50_ value of 19.47 μM.

## 1. Introduction

*Ganoderma* fungi (Ganodermataceae) have long been used as medicines in China, Japan and other Asian countries [1]. Pharmacological investigations revealed that *Ganoderma* extracts or compounds possess antitumor [2], immunomodulatory [3], antioxidant [4], and anti-inflammatory properties [5]. Mounting chemical investigations have been underwent on *Ganoderma* in the past decades which mainly focused on the isolation of triterpeniods and polysaccharides. In 2013, we identified a pair of novel meroterpenoids named lingzhiols with a 5/5/6/6 ring system from *Ganoderma lucidum* as a p-Smad3 inhibitor [6], which received great attention in the related scientific community. So far, more than 150 *Ganoderma* meroterpenoids have been reported from *Ganoderma* species, of which more than two thirds were characterized by us. These structurally diverse meroterpenoids were disclosed to have anti-fibrotic [7,8], neuroprotective [9], antiinflammatory, and antioxidant properties [10,11,12,13,14,15,16]. As a continuation of our research on *Ganoderma* species, *Ganoderma cochlear* was investigated which resulted in the isolation of cochlearines A and B, cochlearoids A–K [8,16], cochlearols A and B [17], and ganocochlearines C–I [18]. These findings inspired us to conduct an additional investigation on this fungus, which therefore led to the isolation of new meroterpenoids, gancochlearol C (**1**), cochlearoid Q (**3**), and gancochlearol D (**2**), together with four known ones, ganomycin C (**4**), ganomycin F (**5**), cochlearol D (**6**), and fornicin D (**7**). In this paper, we describe their isolation, structural elucidation as well as biological activities toward human lung cancer cells (H1975, PC9, A549) and *N*-acetyltransferase.

## 2. Results and Discussion

### 2.1. Structure Elucidation of the Compounds

**Figure 1:**
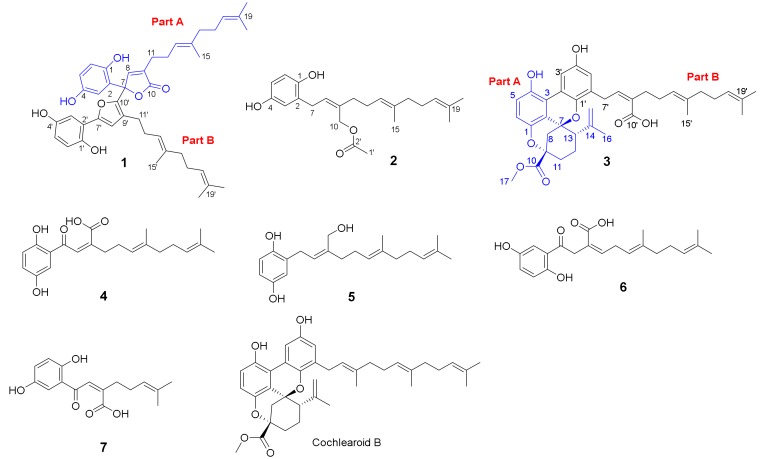
The structures of compounds **1**–**7** from *Ganoderma cochlear*.

**Figure 2:**
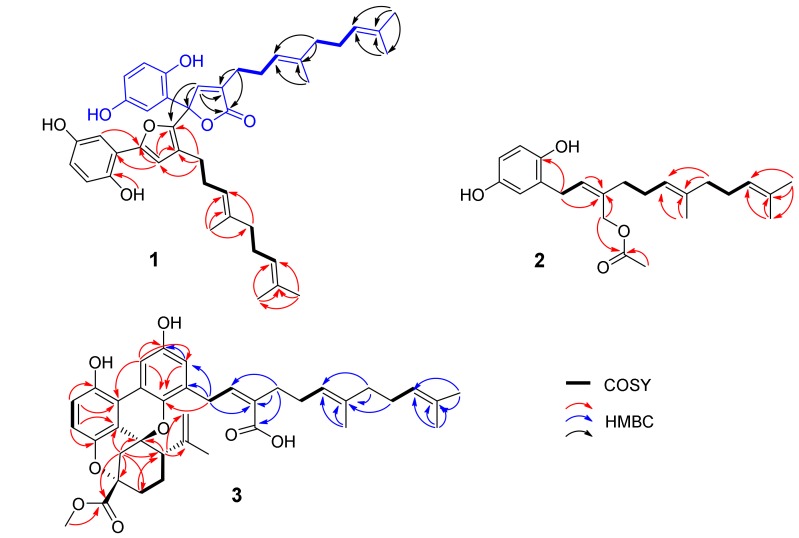
Key ^1^H-^1^H COSY and HMBC correlations for (**1**–**3**).

**Figure 3:**
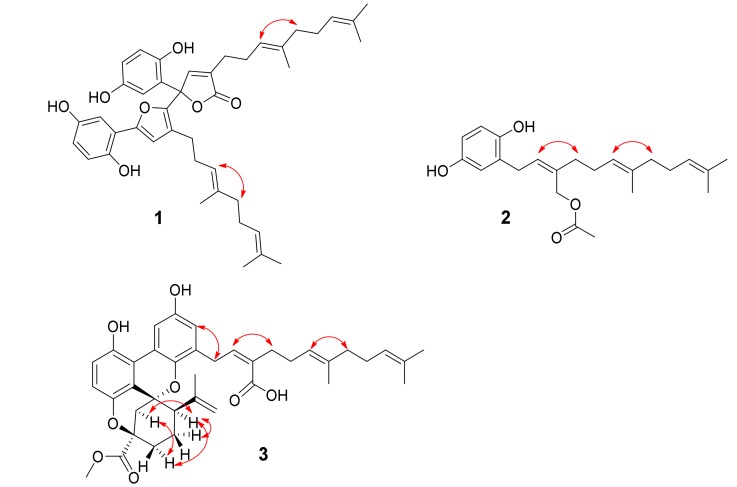
Key ROESY correlations for **1**–**3**.

Gancochlearol C (**1**), obtained as a yellow gum, has a molecular formula C_42_H_50_O_7_ (eighteen degrees of unsaturation) on the basis of its HREIMS at *m*/*z* 666.3565 [M]^+^ (calcd for 666.3557), ^13^C-NMR, and DEPT spectra. The ^1^H-NMR spectrum of **1** (Table 1) exhibits two typical ABX spin systems (δ_H_ 6.84, d, *J* = 2.2 Hz, H-3′; δ_H_ 6.61, d, *J* = 8.5 Hz, H-6′; δ_H_ 6.57, dd, *J* = 8.5, 2.2 Hz, H-5′; δ_H_ 6.79, d, *J* = 2.5 Hz, H-3; δ_H_ 6.69, d, *J* = 8.7 Hz, H-6; δ_H_ 6.49, dd, *J* = 8.7, 2.5 Hz, H-5). The ^13^C-NMR and DEPT spectra show that this substance contains 42 carbons, including six methyls, eight methylenes, twelve methines, and fifteen quaternary carbons (including one carbonyl). These signals suggest that **1** is likely a meroterpenoid dimer when compared with the NMR data of previously reported meroterpeniods from this genus [6,7,8,9,10,11,12,13,14,15,16,17,18]. When careful interpretation of the NMR data, we found that this compound contains two parts (C-1–C-21 and C-1′–C-21′; Figure 1). The data of part A is very similar to those of ganomycin I [19], differing in that C-7 is a quaternary carbon in **1** with a downfield shift at δ_C_ 84.6. The HMBC correlations of H-3, H-8/C-7, H-8/C-9 (δ_C_ 130.7), C-10 (δ_C_ 172.4) support the structure of part A as shown. In the same manner as that of part A, the structure of part B is similar to that of deoxyshikonofuran [20]. One difference between part B and deoxyshikonofuran is that a 10-carbon side chain is replaced by a sesquiterpenoid residue in **1**. In addition, C-10′ is a quaternary carbon in **1**. The structure of part B was further confirmed by 2D-NMR experiments. The ^1^H–^1^H COSY spectrum of **1** shows correlations of H-11′/H-12′/H-13′, H-16′/H-17′/H-18′ (Figure 2, bold lines). The HMBC spectrum (Figure 2) gives correlations of H_3_-20′, H_3_-21′/C-18′ (δ_C_ 124.1), C-19′ (δ_C_ 130.7), H_3_-20′/C-21′, H_3_-15′, H-16′/C-13′ (δ_C_ 123.5), C-14′ (δ_C_ 135.0), H_3_-15′/C-16′ (δ_C_ 39.1), and H-12′/C-14′. These correlations indicate the presence of a side chain in **1** consisting of two isoprenyl moieties (Figure 2). Further HMBC correlations of H-8′ (δ_H_ 6.76), H-11′ (δ_H_ 2.12)/C-9′ (δ_C_ 124.7), C-10′ (δ_C_ 143.1) indicate another isoprenyl residue in the structure. There are two possible connections between parts A and B, which are C-7–C-10′ or C-7–C-8′. The observation of HMBC correlations of H-8′/C-2′ (δ_C_ 117.2), H-8/C-10′ (δ_C_ 143.1) together with the chemical shifts of C-8′ (δ_C_ 112.8) and C-10′ indicate that C-7 is connected to C-10′. As for the geometry of **1**, the ROESY correlations (Figure 3) of H-12/H-15 and H-12′/H-15′ indicate that both ∆^13(14)^ and ∆^13′(14′)^ double bonds are *E* configuration. Of note, **1** was isolated as a racemic mixture. Further separation by chiral phase HPLC afforded (+)-**1** and (−)-**1**. By comparison CD curves of (−)-**1** with those in literature [21], the absolute configuration for **1** was thus assigned as 7R for (−)-**1** and 7S for (+)-**1**. Thus far, the structure of **1** was assigned.

Gancochlearol D (**2**), isolated as a brownish yellow gum, has the molecular formula C_23_H_32_O_4_ (8 degrees of unsaturation), deduced from analyses of its HRESIMS at *m*/*z* 371.2199 [M − H]^−^ (C_23_H_31_O_4_ calcd for 371.2228), ^13^C-NMR, and DEPT spectra. The ^1^H-NMR spectrum (Table 2) exhibits an ABX spin system (δ_H_ 6.58 (1H, d, *J* = 8.5 Hz, H-6), 6.52 (1H, d, *J* = 2.9 Hz, H-3), 6.45 (1H, dd, *J* = 8.5, 2.9 Hz, H-5)). The ^13^C-NMR and DEPT spectra display four methyl, six methylene, six olefinic methine, seven quaternary carbons (including a carbonyl). These signals are very similar to those of ganomycin F (**5**) [22], differing in that an acetyl group is located at C-10 in **2**, which is not the case for ganomycin F (**5**). This conclusion is confirmed by the HMBC correlations of H-10, H_3_-1′/C-2′ (δ_C_ 173.0). The ROESY correlation of H-8/H-11 indicates the Z configuration of the ∆^8(9)^ double bond (Figure 3). Whereas, the ROESY correlation of H-12/H-15 indicates that the ∆^13(14)^ double bond is *E* configuration (Figure 3). Thus, the structure of **2,** named gancochlearol D, was determined to be that shown in Figure 1.

Cochlearoid Q (**3**) has the molecule formula C_38_H_44_O_8_ as assigned by using a combination of HRESIMS at *m/z* 629.3098 [M + H]^+^ (calcd for C_38_H_45_O_8_, 629.3109), ^13^C-NMR and DEPT spectroscopic data, indicating that it contains 17 degrees of unsaturation. The ^1^H-NMR spectrum (Table 3) of **3** suggests the presence of a 1,2,3,4-tetrasubstituted benzene ring evidenced by the observation of an AB system (δ_H_ 6.76 (1H, d, *J* = 8.8 Hz, H-6), 6.72 (1H, d, *J* = 8.8 Hz, H-5)). Additional aromatic proton signals at δ_H_ 7.90 (1H, d, *J* = 2.9 Hz H-3′) and δ_H_ 6.47 (1H, d, *J* = 2.9 Hz, H-5′) suggest that this compound also has a 1,3,4,5-tetrasubstituted benzene ring. The ^13^C-NMR and DEPT spectra of **3** show 38 carbons attributed to five methyls, ten methylenes, eigtht methines, and fifteen quaternary carbons. These data are similar to those of cochlearoid B [12], indicating that they are analogues. The only difference between **3** and cochlearoid B is that C-10′ in **3** is oxidized to a carboxylic acid (δ_C_ 172.0) rather than a methyl in cochlearoid B supported by an HMBC correlation of H-8′ (δ_H_ 5.95)/C-10′. In the ROESY spectrum, the correlations of H-13/Ha-8 (δ_H_ 2.47), Hb-11 (δ_H_ 1.97), and Ha-8/Hb-11 indicate that the relative configuration of **3** is same as that of cochlearoid B. The geometry of the ∆^8′^^(^^9′^^)^ and ∆^13′(14′)^ double bonds were respectively assigned as *Z* and *E*-forms supported by the observed ROESY correlations of H-8′/H-11′ and H-13′/H-15′ (Figure 3). It is note that **3** was isolated as a racemic mixture. Further separation by chiral HPLC afforded (+)-**3** and (−)-**3**, whose absolute configurations were further determined by CD comparison with those of previously reported data [12]. The CD spectrum of (+)-**3** is similar to that of (+)-cochlearoid B, suggesting that they have identical absolute configurations at their stereogenic centers. The absolute configuration of **3** was thus assigned as 7*R*,9*S*,13*S* for (+)-**3** and 7*S*,9*R*,13*R* for (−)-**3**, respectively. As a consequence, compound **3**, named cochlearoid Q, was determined.

The known compounds were identified as ganomycin C (**4**) [23], ganomycin F (**5**) [22], cochlearol D (**6**) [23], and fornicin D (**7**) [22], respectively, by comparison their spectroscopic data with those reported in the literature.

### 2.2. Biological Evaluation

All the isolated meroterpenoids were evaluated for their cytotoxic activity against three human lung cancer cell lines (H1975, PC9, A549). The results show that compounds **2** and **5** are cytotoxic against A549 cells at 40 μM, with **5** shown to be the most active (Figure S46). Besides, **2** and **5** are also found to be active against H1975 and PC9 cells at 40 μM. With this, the cytotoxic potency of **2** and **5** toward three cancer cell lines were further determined using the CCK-8 assay. As shown in Table 4, compound **2** inhibits three cancer cells (H1975, PC9, A549) with IC_50_ values of 32.43, 40.57, and 30.65 μM, respectively. However, compound **5** appears to be more potent than **2** with IC_50_ values of 19.47, 35.70, and 21.60 μM, respectively, against H1975, PC9, and A549 cells. In this assay, erlotinib was used as the positive control with the IC_50_ values of 7.66, 0.085, and 4.59 μM, respectively, toward the above mentioned cells.

**Figure 4:**
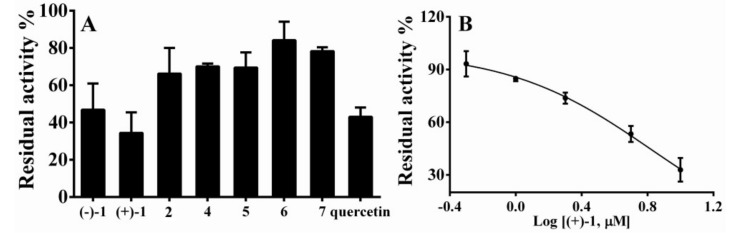
NAT2 inhibition of the compounds.

Arylamine *N*-acetyltransferase (NAT) was reported to be related with synthesis of mycolic acids and complex lipids in *Mycobacterium bovis*, which makes NAT a novel drug target for tuberculosis [24]. With this, all the isolates except for compounds (+)-**3** and (−)-**3** were tested for their inhibitory activity toward the recombinant NAT2 isoform. It was found that (+)-**1** and (−)-**1** could inhibit NAT2 at 10 μM, and (+)-**1** is more potent than quercetin, which was the positive control used in this study. Based on this observation, a subsequent experiment was carried out to reveal that (+)-**1** has an IC_50_ value of 5.29 ± 0.10 μM toward NAT2 (Figure 4).

## 3. Experimental Section

### 3.1. General Procedures

Column chromatography was undertaken on silica gel (200–300 mesh, Qingdao Marine Chemical Inc., Qingdao, China), MCI gel CHP 20P (75–150 μm, Mitsubishi Chemical Industries, Tokyo, Japan), RP-18 (40–60 µm; Daiso Co., Tokyo, Japan), and Sephadex LH-20 (Amersham Pharmacia, Uppsala, Sweden). Optical rotations were measured on a Bellingham + Stanley ADP 440 + digital polarimeter (Bellingham & Stanley, Kent, UK). UV spectra were obtained on a Shimadzu UV-2600 spectrometer (Shimadzu Corporation, Tokyo, Japan). CD spectra were measured on a Chirascan instrument (Agilent Technologies, Santa Clara, CA, USA). Semi-preparative or analytic HPLC was carried out using an Agilent 1200 liquid chromatograph (Agilent Technologies, Santa Clara, CA, USA). The column used was a YMC-Pack ODS-A 250 mm × 9.4 mm, i.d., 5 µm, or a Thermo Hypersil GOLD-C18 250 mm × 21.2 mm, i.d., 5 µm. NMR spectra were recorded at room temperature on a Bruker AV-400 or a Bruker Avance III 600 spectrometer (Bruker, Karlsruhe, Germany) with TMS as an internal standard. EIMS and HRESIMS data of compound **1** were collected by an AutoSpec Premier P776 spectrometer (Waters Corporation, Milford, MA, USA). ESIMS and HRESIMS data of **2** and **3** were collected by a Shimazu LC-20AD AB SCIEX triple TOF 5600+ MS spectrometer (Shimadzu Corporation, Tokyo, Japan). ESIMS of **4** was collected by an Agilent G6230TOF MS spectrometer (Agilent Technologies). ESIMS of **5**–**7** were collected on an Agilent G6125B LC/MSD spectrometer (Agilent Technologies).

### 3.2. Fungal Material

The fruiting bodies of *Ganoderma cochlear* were purchased from Tongkang Pharmaceutical Co. Ltd. in Guangzhou, Guangdong Province, China, in July 2014. The material was authenticated by Prof. Zhu-Liang Yang at Kunming Institute of Botany, Chinese Academy of Sciences, Kunming, China, and a voucher specimen (CHYX-0589) has been deposited at School of Pharmaceutical Sciences, Shenzhen University Health Science Center, Shenzhen, China, since October 2017.

### 3.3. Extraction and Isolation

The powders of *Ganoderma cochlear* (200 kg) fruiting bodies were extracted with 80% EtOH under reflux (3 × 120 L, 4, 3, 3 h) and concentrated under reduced pressure to yield a crude extract. An aliquot (8 kg extract corresponding to 95 kg fungal material) was suspended in water and partitioned with EtOAc thrice, followed by removal of solvents to afford an EtOAc soluble extract (4 kg). The extract was separated by silica gel column with increasing acetone in petroleum ether to provide four parts (Fr.1–Fr.4). Fr.2 (860 g) was further divided into six parts (Fr.2.1–Fr.2.6) by MCI gel CHP 20P column (MeOH/H_2_O, 60–100%). Fr.2.4 (107 g) was submitted to a silica gel column eluted with increasing EtOAc in petroleum ether (10:1–0:100) to afford five parts (Fr.2.4.1–Fr.2.4.5). Among them, Fr.2.4.4 (40 g) was cut into three portions (Fr.2.4.4.1–Fr.2.4.4.3) by RP-18 (MeOH/H_2_O, 60–100%). Of which, Fr.2.4.4.2 (25 g) was separated by a silica gel column eluted with increasing acetone in petroleum ether (8:1–0:100) to afford four parts (Fr.2.4.4.2.1–Fr.2.4.4.2.5). Fr.2.4.4.2.2 (8 g) was purified by Sephadex LH-20 (MeOH) followed by preparative HPLC (MeOH/H_2_O, 85%) to get five fractions (Fr.2.4.4.2.2.1–Fr.2.4.4.2.2.5). Of which, Fr.2.4.4.2.2.1 (500 mg) was purified by semi-preparative HPLC (MeOH/H_2_O, 85%) to get compounds **1** (15.2 mg, t_R_ = 28.0 min) and **3** (12.6 mg, t_R_ = 28.8 min), and **4** (10.4 mg, t_R_ = 30.7 min). Fr.2.4.4.2.1 (1 g) was purified by Sephadex LH-20 (MeOH) followed by semi-preparative HPLC (MeCN/H_2_O, 68:32) to get compound **2** (10.7 mg, t_R_ = 20.6 min), **5** (20.1 mg, t_R_ = 24.5 min), and **6** (15.6 mg, t_R_ = 26.8 min).

Fr.2.1 (120 g) was separated by a RP-18 column (MeOH/H_2_O, 35–100%) to provide five parts (Fr.2.1.1–Fr.2.1.5). Of which Fr.2.1.1 (5.8 g) was submitted to Sephadex LH-20 (MeOH) to get three portions (Fr.2.1.1.1–Fr.2.1.1.3). Among them, Fr.2.1.1.3 (500 mg) was separated by preparative thin layer chromatography (PTLC) (CHCl_3_/MeOH, 7:1) (Rf = 0.6) followed by Sephadex LH-20 (MeOH) filtration to get compound **7** (25.4 mg).

Compounds **1** and **3**, which are racemic, were further purified by HPLC on chiral phase (Daicel Chiralpak IC, flow rate: 3 mL/min) to afford enantiomers [(+)-**1** (3.5 mg, t_R_ = 15.8 min) and (−)-**1** (4.7 mg, t_R_ = 20.2 min) (*n*-hexane/ethanol, 90:10); (+)-**3** (6.0 mg, t_R_ = 12.5 min) and (−)-**3** (5.8 mg t_R_ = 20.2 min) (*n*-hexane/ethanol/CF_3_COOH, 90:10:0.01%).

### 3.4. Compound Characterization Data

*Gancochlearol C* (**1**): Brownish yellow gum; [α]_D_^29^ + 5.6 (*c* 0.30, MeOH); CD (MeOH), Δε_198_ − 0.65, (+)-**1**; [α]_D_^29^ − 10.8 (*c* 0.30, MeOH); CD (MeOH) Δε_201_ + 4.66, (−)-**1**; UV (MeOH) λ_max_ (logε) 194 (4.23), 279 (3.65), 327 (3.62) nm; EIMS *m*/*z* 666 [M]^+^, HREIMS 666.3565 (calcd for C_42_H_50_O_7_, 666.3557); ^1^H- and ^13^C-NMR data, see Table 1.

*Gancochlearol D* (**2**): Brownish yellow gum; UV (MeOH) λ_max_ (logε) 206 (4.07), 295 (3.29) nm; ESIMS *m*/*z* 371 [M − H]^−^, HRESIMS *m*/*z* 371.2199 [M − H]^−^, (calcd for C_23_H_31_O_4_, 371.2228); ^1^H- and ^13^C-NMR data, see Table 2.

*CochlearoidQ* (**3**): Brownish yellow gum; [α]_D_^2^^9^ + 35.8 (*c* 0.22, MeOH); CD (MeOH), Δε_211_ + 2.98, Δε_240_ + 13.63, Δε_278_ − 2.16; Δε_325_ + 1.91 (+)-**3**; [α]_D_^2^^9^ − 45.7 (*c* 0.18, MeOH); CD (MeOH) Δε_214_ − 0.77, Δε_242_ − 13.76, Δε_279_ + 3.37, Δε_323_ − 0.45; (−)-**3**; UV (MeOH) λ_max_ (logε) 213 (4.05), 229 (3.97), 337 (3.38) nm; ESIMS *m*/*z* 629 [M + H]^+^, HRESIMS *m*/*z* 629.3098 [M + H]^+^ (C_38_H_45_O_8_ calcd for 629.3109); ^1^H- and ^13^C-NMR data, see Table 3.

### 3.5. Cell Viability

The human NSCLC cell line (NCL-H1975) was purchased from Shanghai Cell Bank of Chinese Academy of Sciences, (Shanghai, China). PC9 and A549 were obtained from American Type Culture Collection (ATCC, Manassas, VA, USA). Cells were cultured in DMEM (Gibco, Grand Island, NY, USA) with 4.5 g/L glucose and 10% FBS (Gibco, Grand Island, NY, USA). Erlotinib used in the experiments were purchased from Selleck (Selleck Chemicals, Houston, TX, USA). Cytotoxicity was determined using the previously described method [25]. Three human lung cancer cell lines were incubated in 96-well plates (5000 cells/well) respectively to attach overnight and then exposed to drug treatments for additional 24 h. And the CCK solution was added, after 2 h of incubation, the absorbance was measured at a wavelength of 450 nm. Cell viability was measured by a TransDetect^®^ Cell Counting Kit (TransGen Biotech, Beijing, China). The IC_50_ values were calculated using the GraphPad Prism 5.0 (GraphPad Software, San Diego, CA, USA).

### 3.6. N-Acetyltransferase (NAT) Inhibition

In order to determine the inhibitory activity of the isolates toward *N*-acetyltransferase (NAT), which plays an important role in the synthesis of mycobacterial cell wall lipids and is thus closely associated with mycobacterial growth, the recombinant NAT2 (Corning Company, Corning, NY, USA, Cat. No. 456282), a key target for the inhibition of *Mycobacterium tuberculosis* was utilized in this study. The inhibition activity of the compounds toward NAT2 were assayed according to the previously reported method [26]. Briefly, the fluorescent probe (CYP1) was incubated with NAT2 in the presence or absence of different compounds including (+)-**1**, (−)-**1**, **2**, and **4**–**7** with the final concentration of 10 μM. After incubation for 30 min, the reaction was terminated by adding acetonitrile and the supernatant was tested by Synergy H1 microplate reader (Bio-Tek, Winooski, VT, USA) with the excitation wavelength at 780 nm. Meanwhile, the positive inhibitor quercetin (10 μM) for NAT2 was also set in this inhibition screening [27]. Finally, the IC_50_ value of compound (+)-**1** was further investigated by adding different concentrations of (+)-**1** (0.5−10 μM) in the above incubation system. Data were fit to log (inhibitor) versus normalized response–variable slope equation using GraphPad Prism 6 (GraphPad Software, San Diego, CA, USA).

## 4. Conclusions

In sum, three new meroterpenoids were characterized from the fruiting bodies of *Ganoderma cochlear* which adds new facets to the structural diversity to the family of *Ganoderma* meroterpenoids. Our findings of cytotoxic meroterpenoids suggest that this class of compounds might justify medicinal applications of *Ganoderma* fungi in cancer. Finally, inhibition of (+)-**1** toward NAT2 implies that it could be a structure template for developing NAT2 inhibitors with therapeutic potential in tuberculosis.

## Figures and Tables

**Table 1:** ^1^H- (600 MHz) and ^13^C-NMR (150 MHz) data of **1** in DMSO-*d*_6_ (δ in ppm, *J* in Hz).

Position	δ_H_	δ_C_	Position	δ_H_	δ_C_
1		146.4	1′		146.7
2		124.3	2′		117.2
3	6.84 (d, 2.2)	111.9	3′	6.79 (d, 2.5)	110.8
4		149.9	4′		149.7
5	6.57 (dd, 8.5, 2.2)	115.8	5′	6.49 (d, 8.7, 2.5)	115.2
6	6.61 (d, 8.5)	116.6	6′	6.69 (d, 8.7)	116.7
7		84.6	7′		148.4
8	7.96 (s)	149.2	8′	6.76 (s)	112.8
9		130.7	9′		124.9
10		172.4	10′		143.1
11	2.30 (m)	24.8	11′	2.12 (m)	24.8
12	2.22 (m)	25.5	12′	1.97 (m)	26.1
13	5.09 (t, 6.5)	122.7	13′	4.98 (overlap)	123.5
14		136.0	14′		135.0
15	1.47 (s)	15.8	15′	1. 49 (s)	15.9
16	1.85 (m)	39.1	16′	1.88 (m)	39.1
17	1.98 (m)	26.2	17′	1.93 (m)	28.5
18	5.04 (t, 6.8)	124.1	18′	4.99 (overlap)	124.1
19		130.7	19′		130.7
20	1.52 (s)	17.7	20′	1.50 (s)	17.6
21	1.60 (s)	25.5	21′	1.59 (s)	25.5
1-OH	9.09 (s)		1′-OH	9.35 (s)	
4-OH	8.86 (s)		4′-OH	8.81 (s)	

**Table 2:** ^1^H- (600 MHz) and ^13^C-NMR (150 MHz) data of **2** in methanol-*d*_4_ (δ in ppm, *J* in Hz).

Position	δ_H_	δ_C_	Position	δ_H_	δ_C_
1		149.0	13	5.10 (t-like, 5.0)	125.0
2		129.1	14		136.4
3	6.52 (d, 2.9)	117.4	15	1. 57 (s)	16.1
4		151.1	16	1.94 (m)	40.8
5	6.45 (dd, 8.5, 2.9)	114.2	17	2.02 (m)	27.7
6	6.58 (d, 8.5)	116.5	18	5.03 (t-like, 6.9)	125.5
7	3.33 (m)	29.3	19		132.0
8	5.57 (t, 7.7)	130.5	20	1.57 (s)	17.8
9		135.2	21	1.65 (s)	25.9
10	4.74 (s)	63.1	1′	2.04 (s)	20.9
11	2.12 (overlap)	36.5	2′		173.0
12	2.12 (overlap)	27.7			

**Table 3:** ^1^H- (600 MHz) and ^13^C-NMR (150 MHz) data of **3** in methanol-*d*_4_ (δ in ppm, *J* in Hz).

Position	δ_H_	δ_C_	Position	δ_H_	δ_C_
1		147.1	1′		146.4
2		119.4	2′		128.8
3		117.5	3′	7.90 (d, 2.9)	114.0
4		149.1	4′		151.3
5	6.76 (d, 8.8)	118.3	5′	6.47 (d, 2.9)	116.6
6	6.72 (d, 8.8)	116.2	6′		123.2
7		76.1	7′	3.83 (dd, 15.7, 7.9)	31.7
8	2.47 (d, 12.5)	41.7		3.66 (dd, 15.7, 7.9)	
	2.33 (dd, 12.5, 2.2)		8′	5.95 (t-like, 7.9)	140.5
9		81.0	9′		133.5
10		173.9	10′		172.0
11	Ha: 2.09 (m)	36.3	11′	2.29 (t, 7.3)	36.1
	Hb: 1.97 (m)		12′	2.15 (m)	28.5
12	Ha: 1.66 (m)	26.7	13′	5.10 (t, 6.9)	124.6
	Hb: 1.47 (m)		14′		136.9
13	2.63 (dd, 13.2, 3.3)	58.2	15′	1.55 (s)	16.2
14		145.7	16′	1.91 (m)	40.7
15	4.46 (s)	114.2	17′	2.10 (m)	27.8
	4.27 (s)		18′	5.04 (t, 6.9)	125.4
16	1.23 (s)	21.1 ^a^	19′		132.0
17	3.80 (s)	53.2	20′	1.56 (s)	17.8
			21′	1.63 (s)	25.9

^a^ The signal was assigned by HSQC.

**Table 4:** Cytotoxic activities of compounds **2** and **5**.

Compound		IC_50_ (μM)	
	H1975	PC9	A549
2	32.43	40.57	30.65
5	19.47	35.70	21.60
Positive control ^a^	7.66	0.085	4.59

^a^ Erlotinib.

## Supplementary Materials

The following are available online. Figures S1–S12: NMR spectra of **1**, Figures S13 and S14: EIMS and HREIMS of **1**, Figures S15 and S16: CD spectrum of **1**, Figures S17–S22: NMR spectra of **2**, Figure S23: HRESIMS of **2**, Figures S24–S29: NMR spectra of **3**, Figures S30 and S31: CD spectrum of **3**, Figures S32: HRESIMS of **3**, Figures S33 and S34: NMR spectra of **4**, Figure S35: ESIMS of **4**, Figures S36 and S37: NMR spectra of **5**, Figure S38: ESIMS of **5**, Figures S39 and S40: NMR spectra of **6**, Figure S41: ESIMS of **6**, Figures S42–S44: NMR spectra of **7**, Figure S45: ESIMS of **7**, Figure S46. Cytotoxicity of the compounds toward three human lung cancer cell lines was measured using TransDetect® Cell Counting Kit.
