# Supplementary material for: Cytotoxic and N-Acetyltransferase Inhibitory Meroterpenoids from Ganoderma cochlear
Source: Molecules. 2018 Jul 20;23(7):1797. doi: 10.3390/molecules23071797 (PMC6100301; doi:10.3390/molecules23071797)
Supplement: Supplementary file 1 [file molecules-23-01797-s001.pdf]

# Supplementary Materials: Cytotoxic and *N*-acetyltransferase inhibitory Meroterpenoids from *Ganoderma cochlear*

Li-Zhi Cheng [1,2,†], Fu-Ying Qin [2,3†], Xiao-Chi Ma[4], Shu-Mei Wang[1], Yong-Ming Yan[2], and Yong-Xian Cheng [1,2,*]

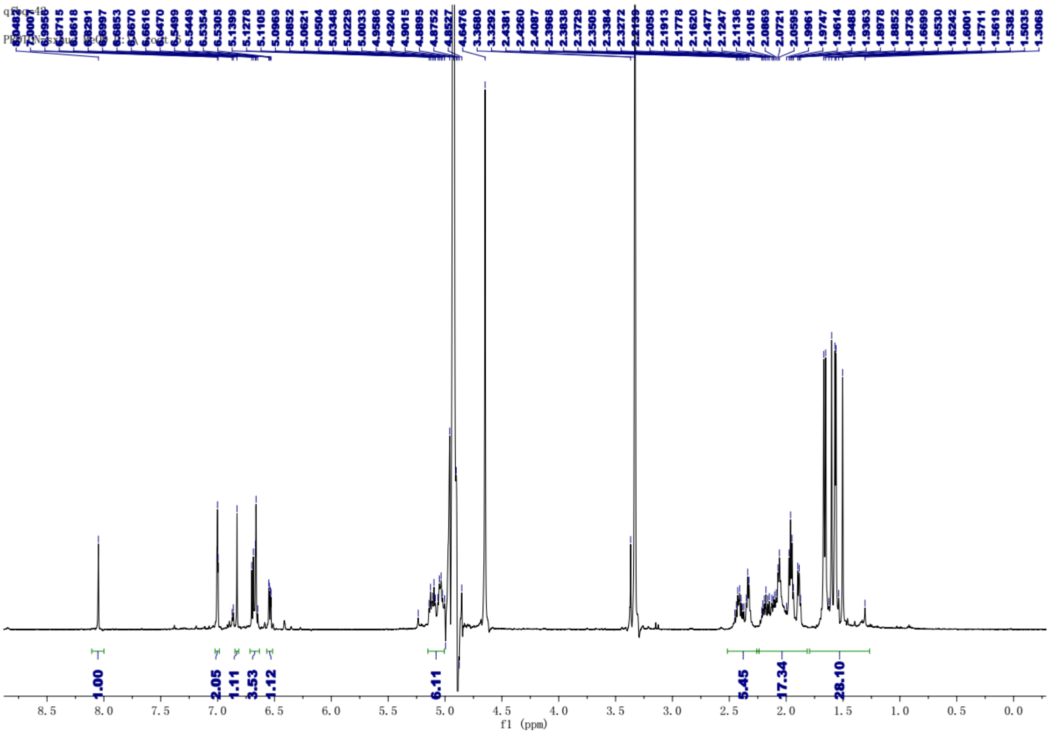


Figure S1.$^{1}$H NMR spectrum of **1** in methanol-*d*$_4$

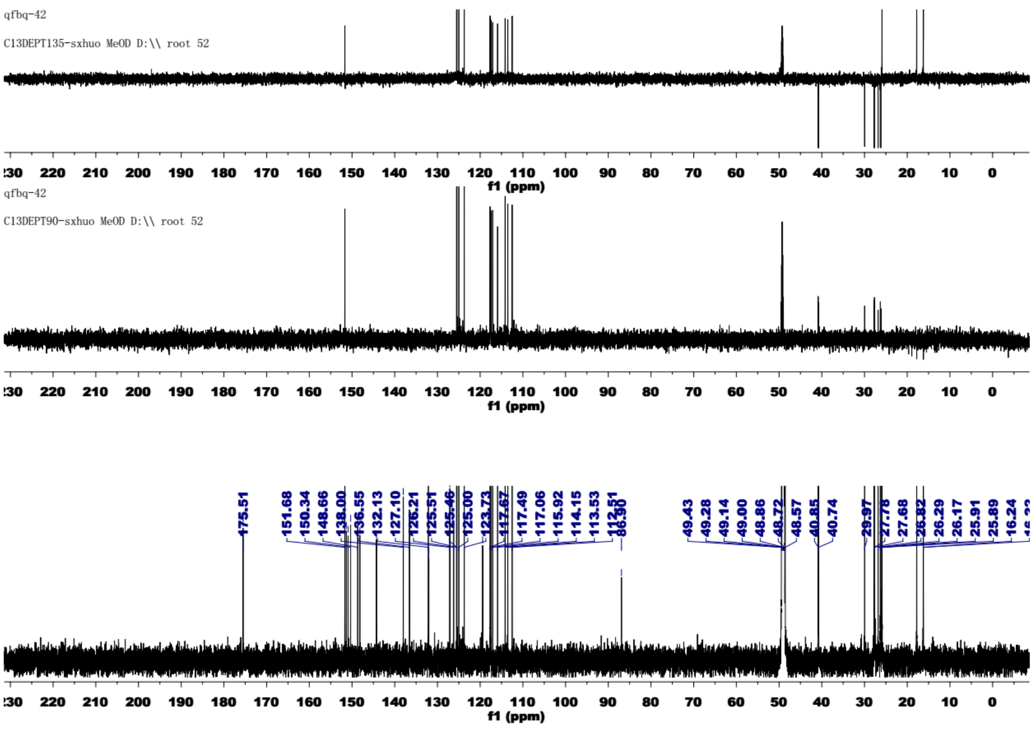


Figure S2. $^{13}$C NMR and DEPT spectra of **1** in methanol-$d_4$

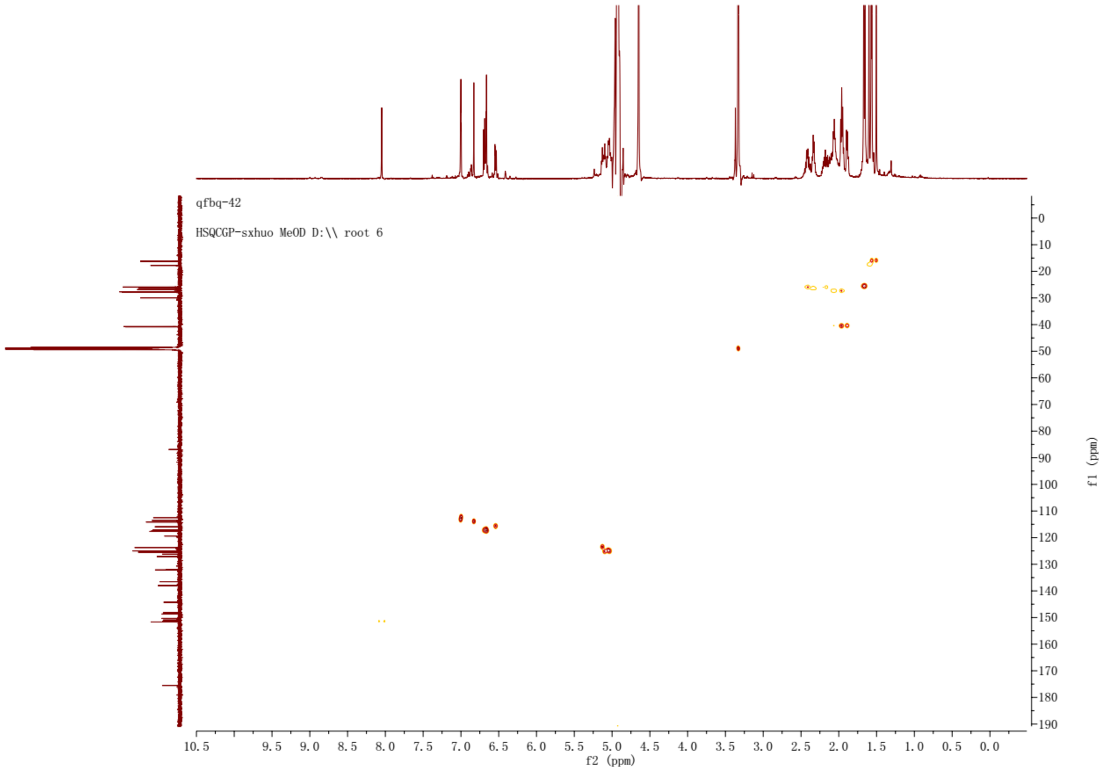


Figure S3. HSQC spectrum of **1** in methanol-$d_4$

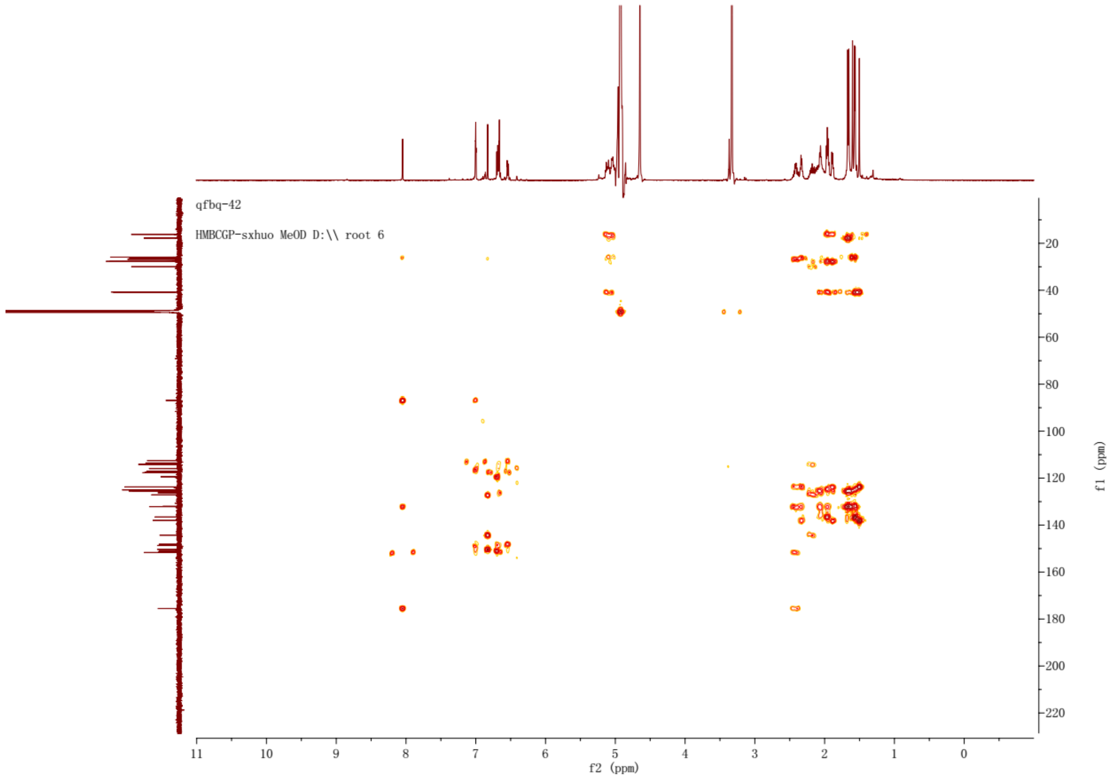


Figure S4. HMBC spectrum of **1** in methanol-$d_4$

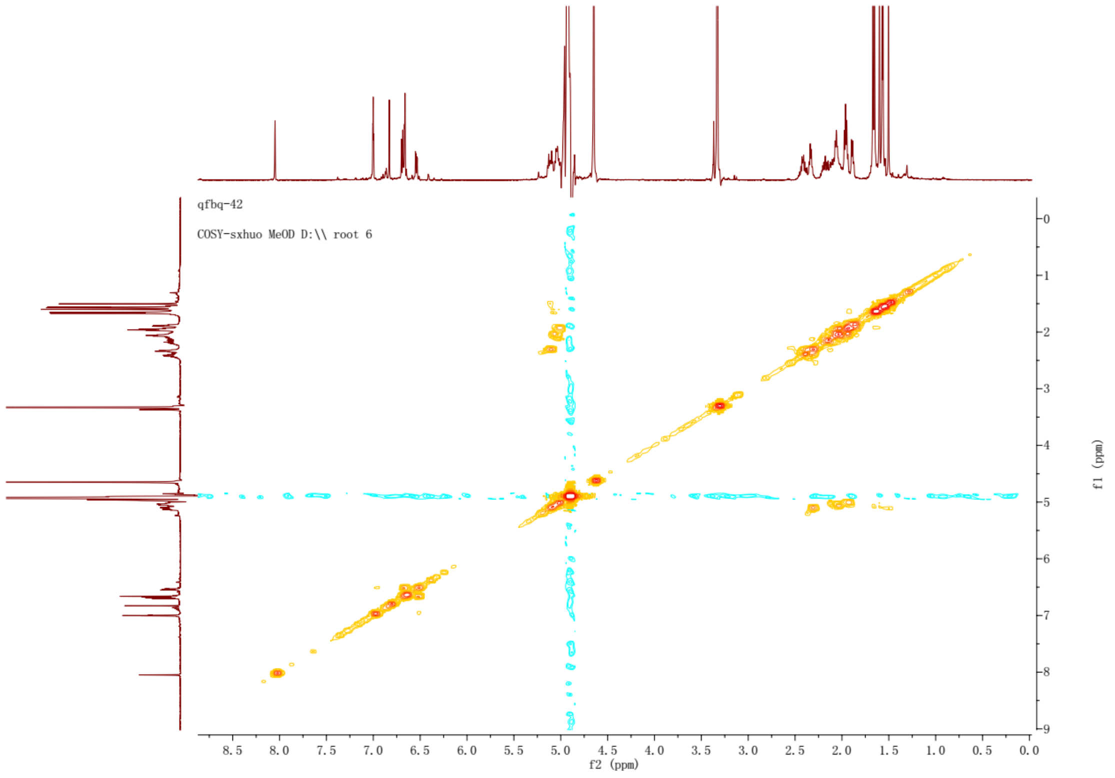


Figure S5. $^1$H-$^1$H COSY spectrum of **1** in methanol-$d_4$

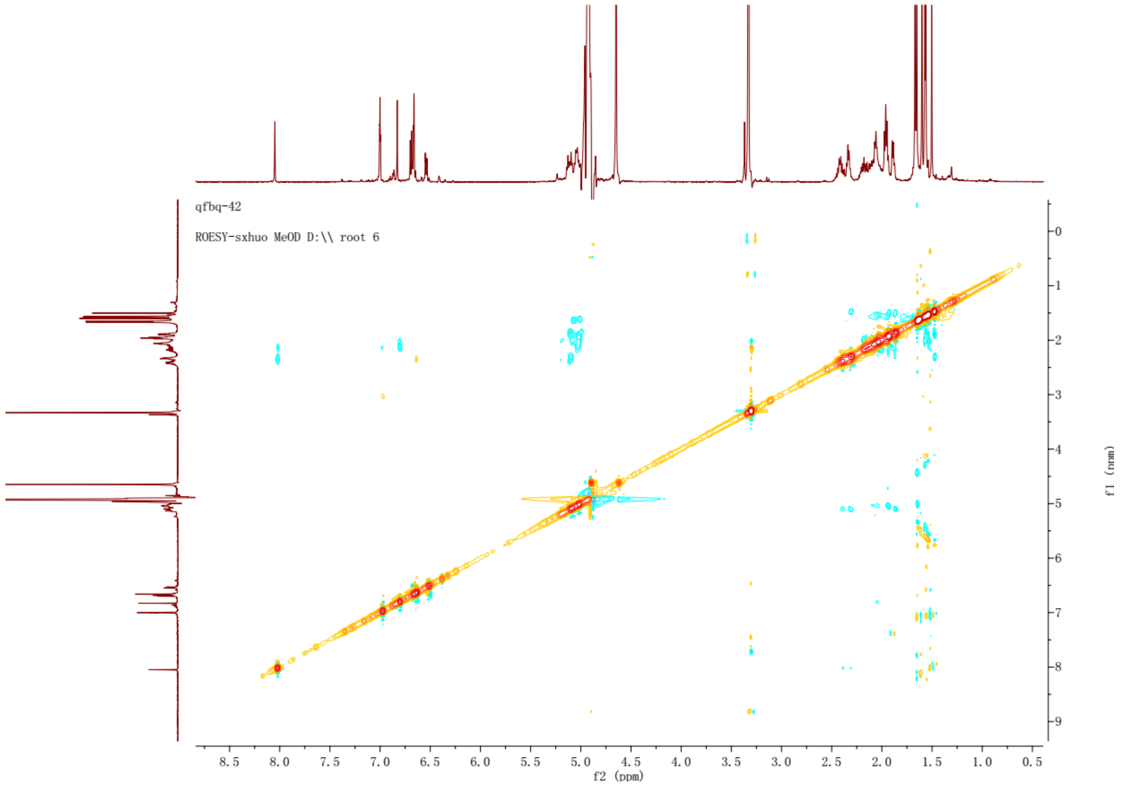


Figure S6. ROESY spectrum of **1** in methanol-$d_4$

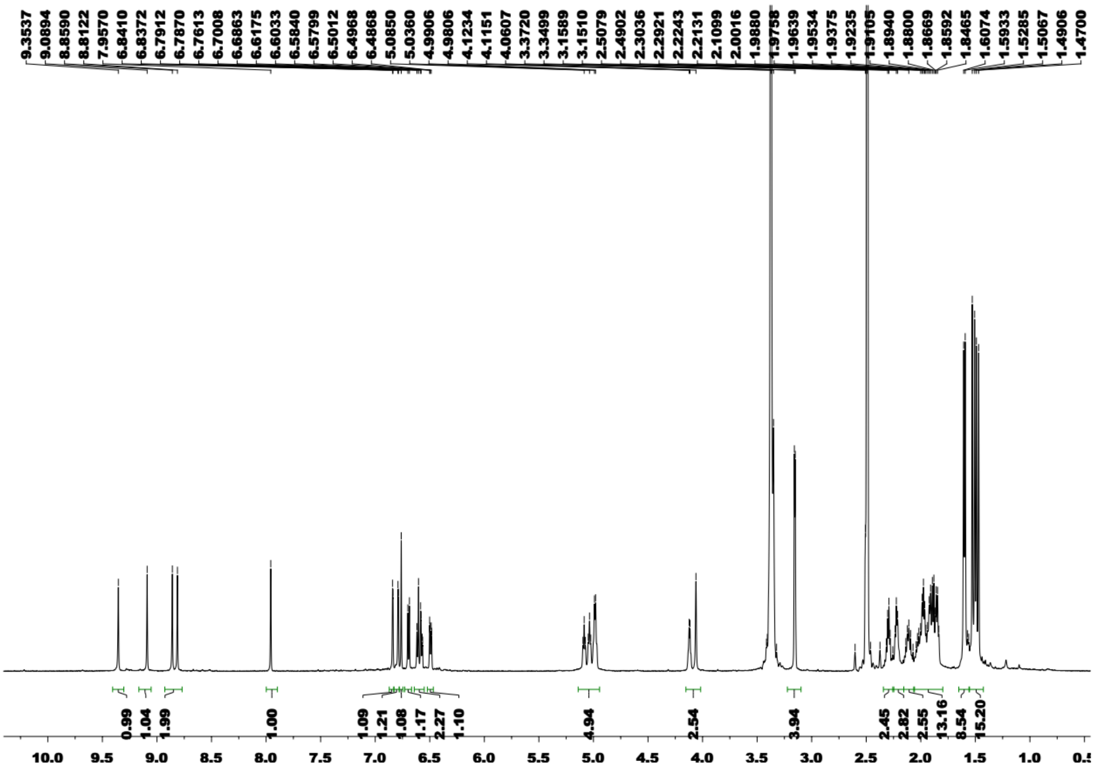

Figure S7. $^1$H NMR spectrum of **1** in DMSO-$d_6$

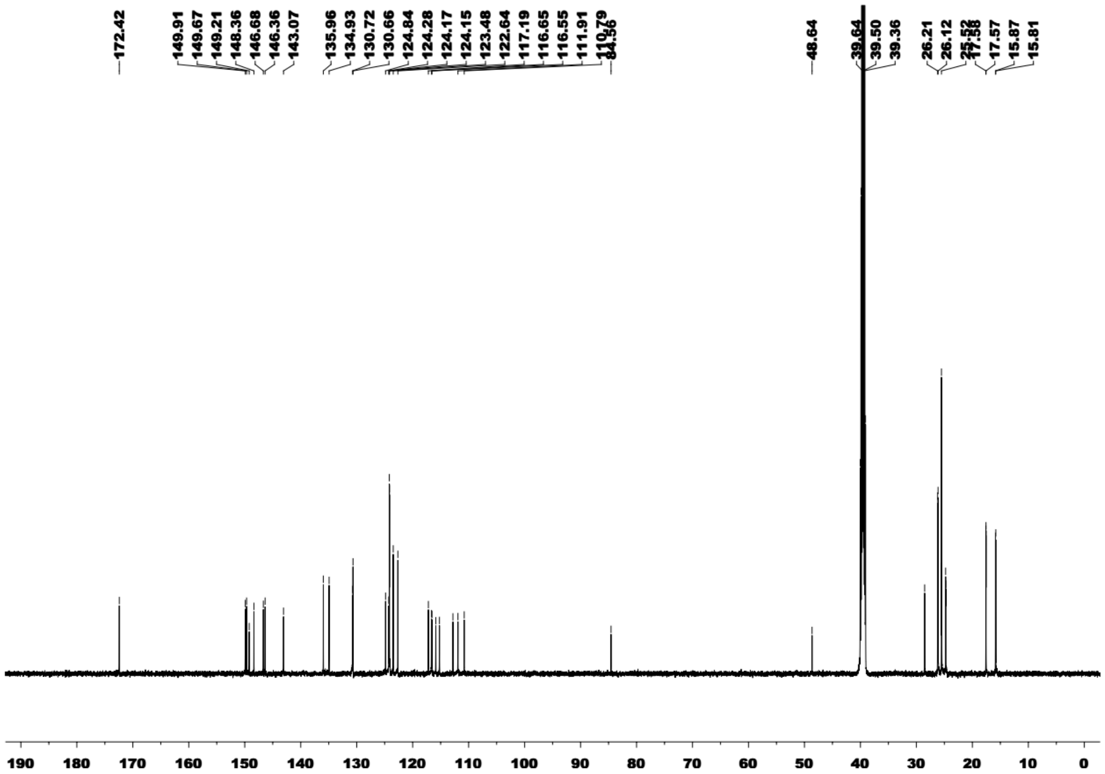


Figure S8. $^{13}$C NMR spectrum of **1** in DMSO-$d_6$

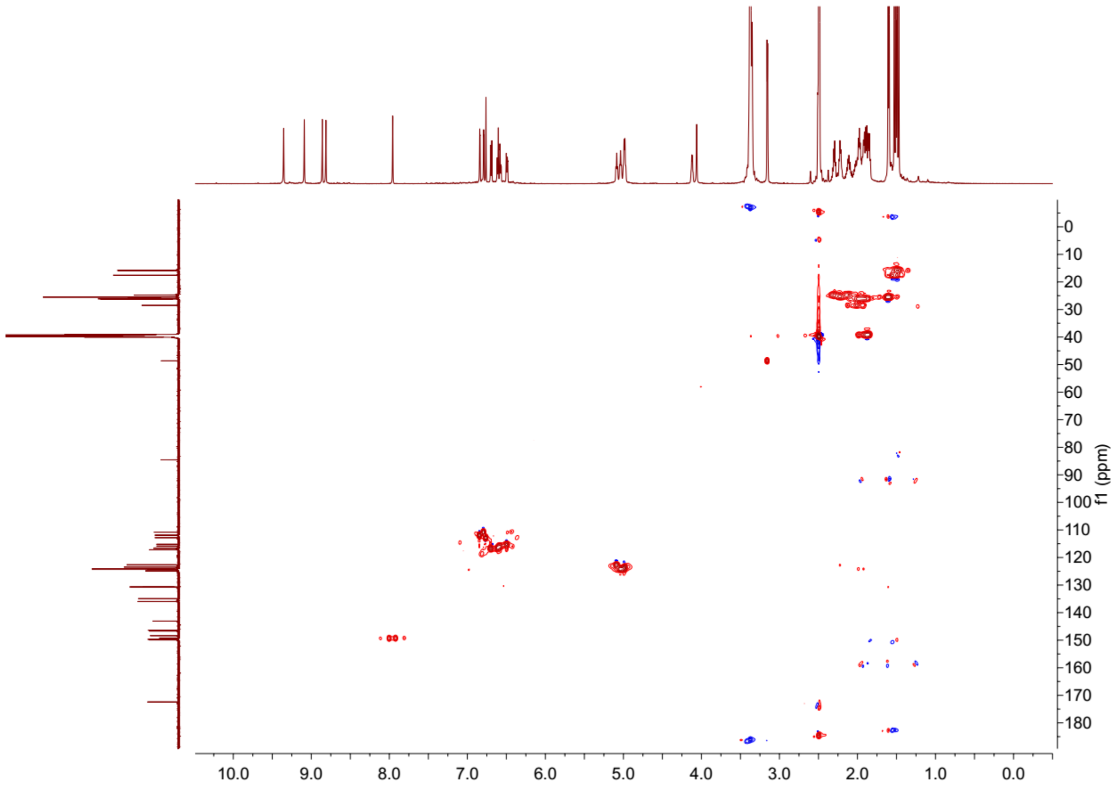


Figure S9. HSQC spectrum of **1** in DMSO-$d_6$

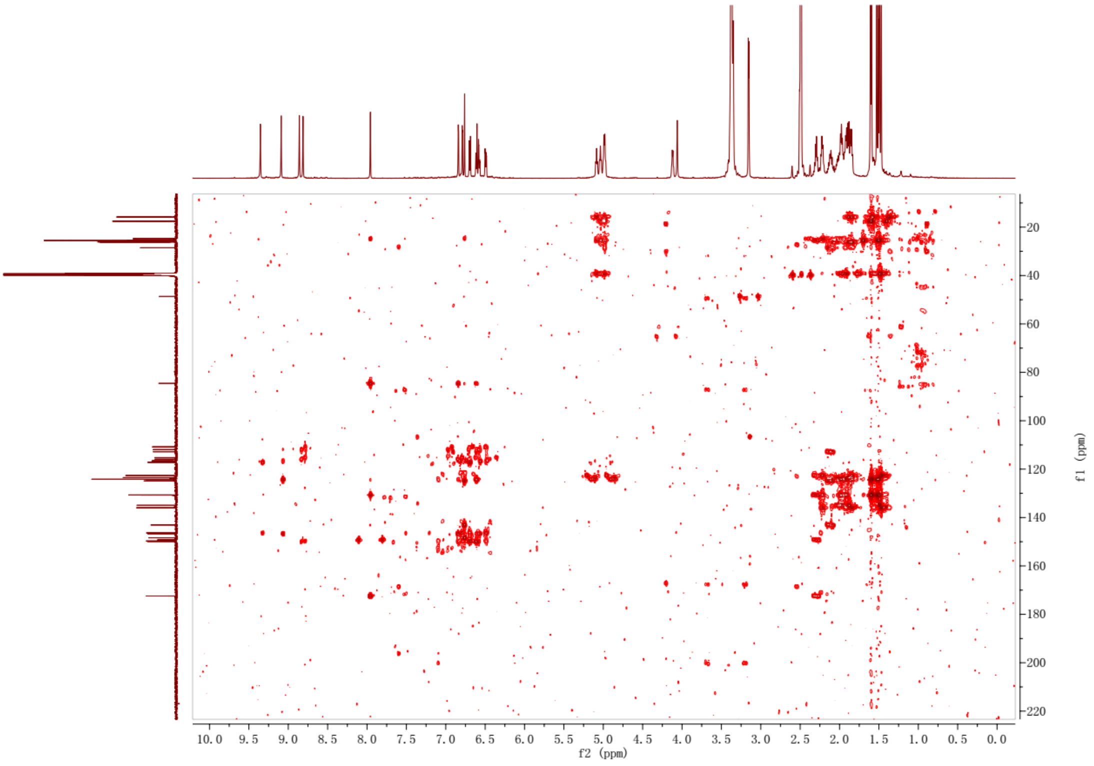


Figure S10. HMBC spectrum of **1** in DMSO-$d_6$

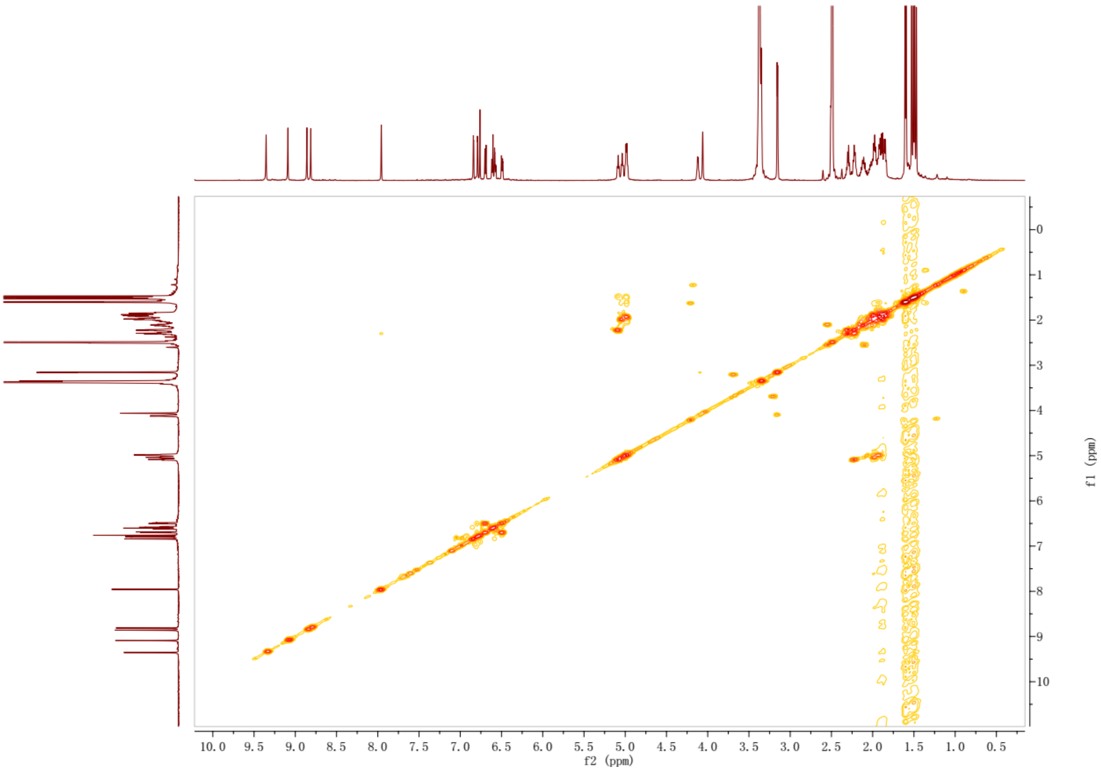


Figure S11. $H^1$-$H^1$ COSY spectrum of **1** in DMSO-$d_6$

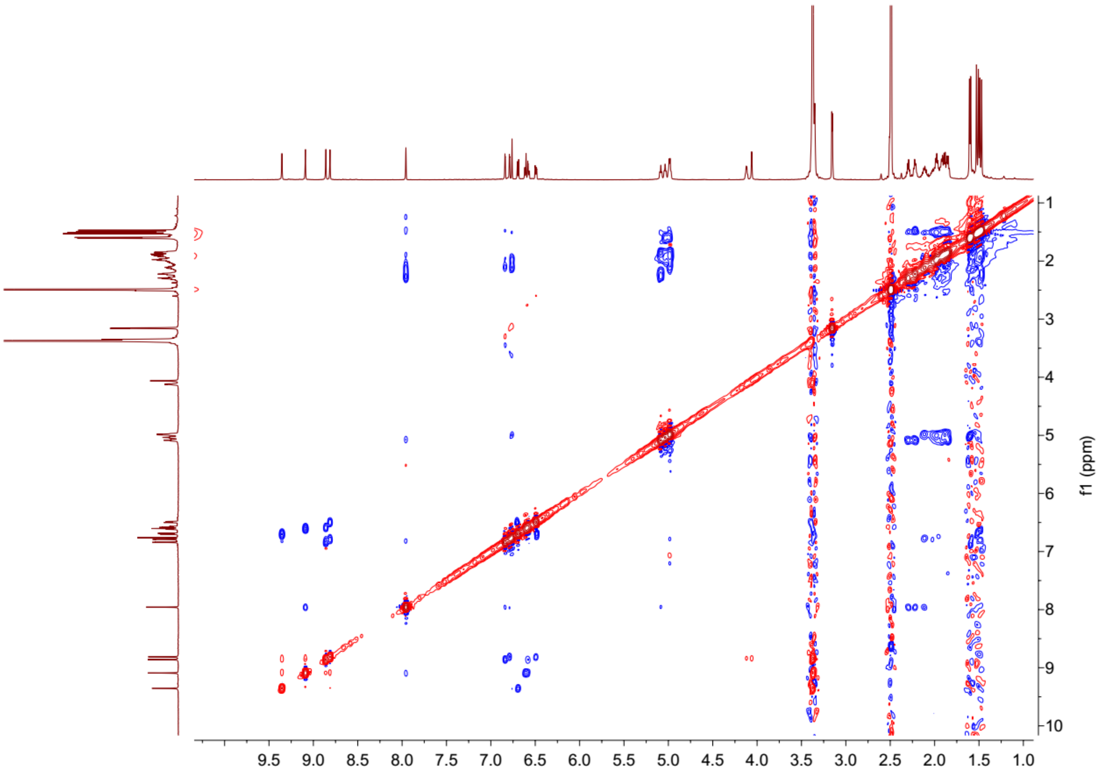


Figure S12. ROESY spectrum of **1** in DMSO-$d_6$

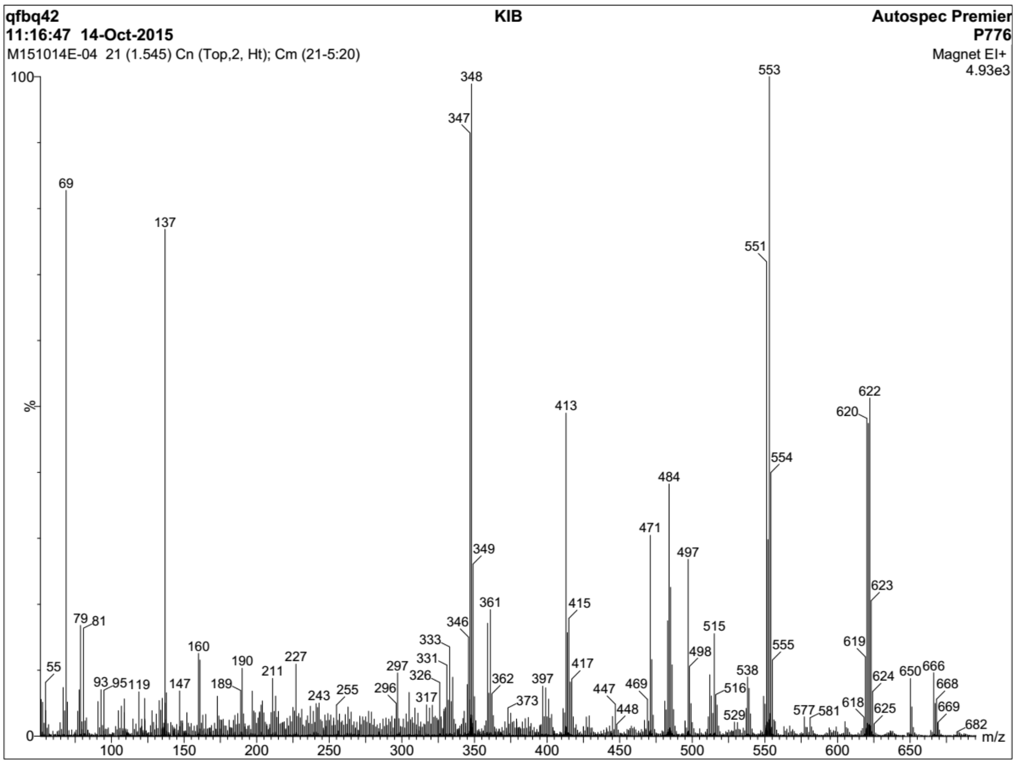


Figure S13. EIMS of **1**

**Elemental Composition Report** 

**Single Mass Analysis**
Tolerance = 10.0 PPM / DBE: min = -10.0, max = 120.0
Selected filters: None

Monoisotopic Mass, Odd and Even Electron Ions
26 formula(e) evaluated with 1 results within limits (up to 51 closest results for each mass)
Elements Used:
C: 0-200 H: 0-400 O: 6-8

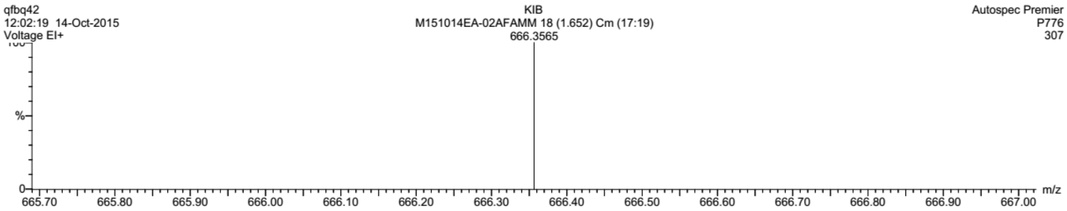


| | | | | | | |
|---|---|---|---|---|---|---|
| Minimum: | | | | -10.0 | | |
| Maximum: | | 200.0 | 10.0 | 120.0 | | |
| Mass | Calc. Mass | mDa | PPM | DBE | i-FIT | Formula |
| 666.3565 | 666.3557 | 0.8 | 1.2 | 18.0 | 5546174.5 | C42 H50 O7 |

Figure S14. HREIMS of **1**

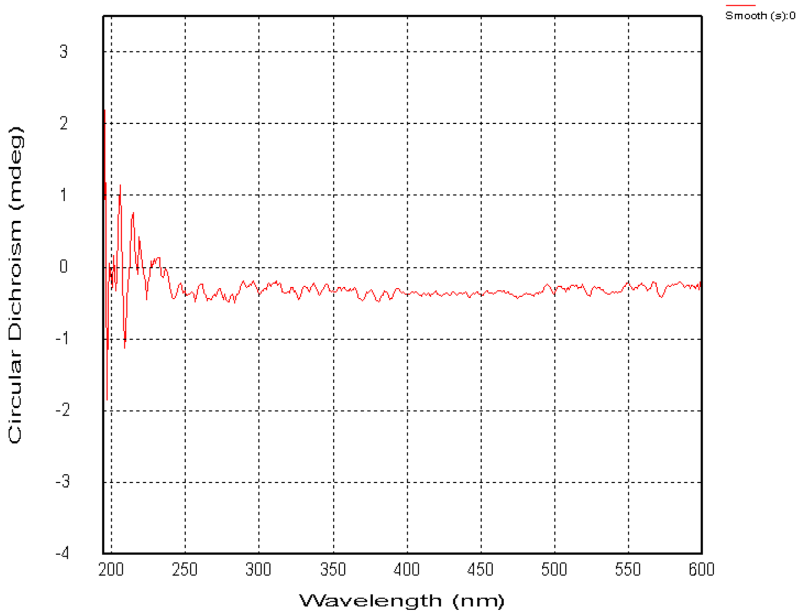


Figure S15 CD spectrum of (+)-**1**

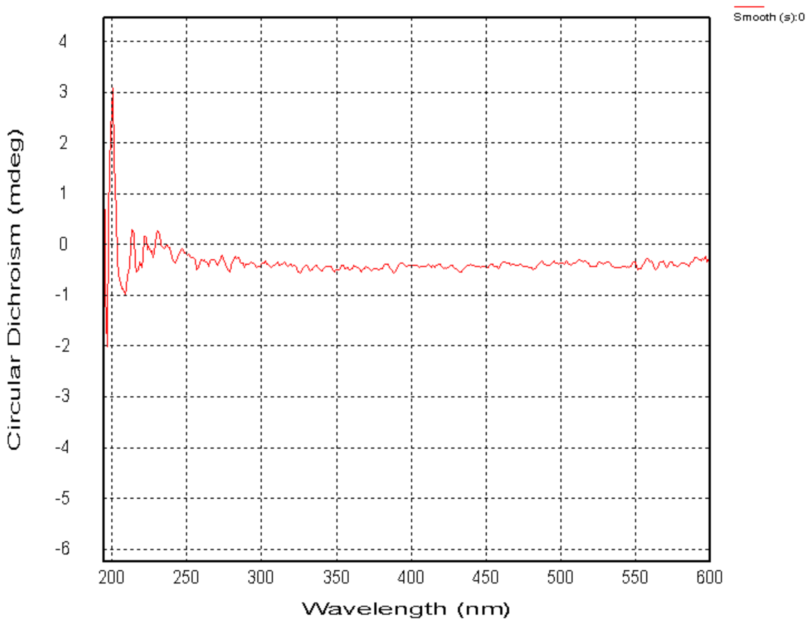


Figure S16 CD spectrum of (–)-**1**

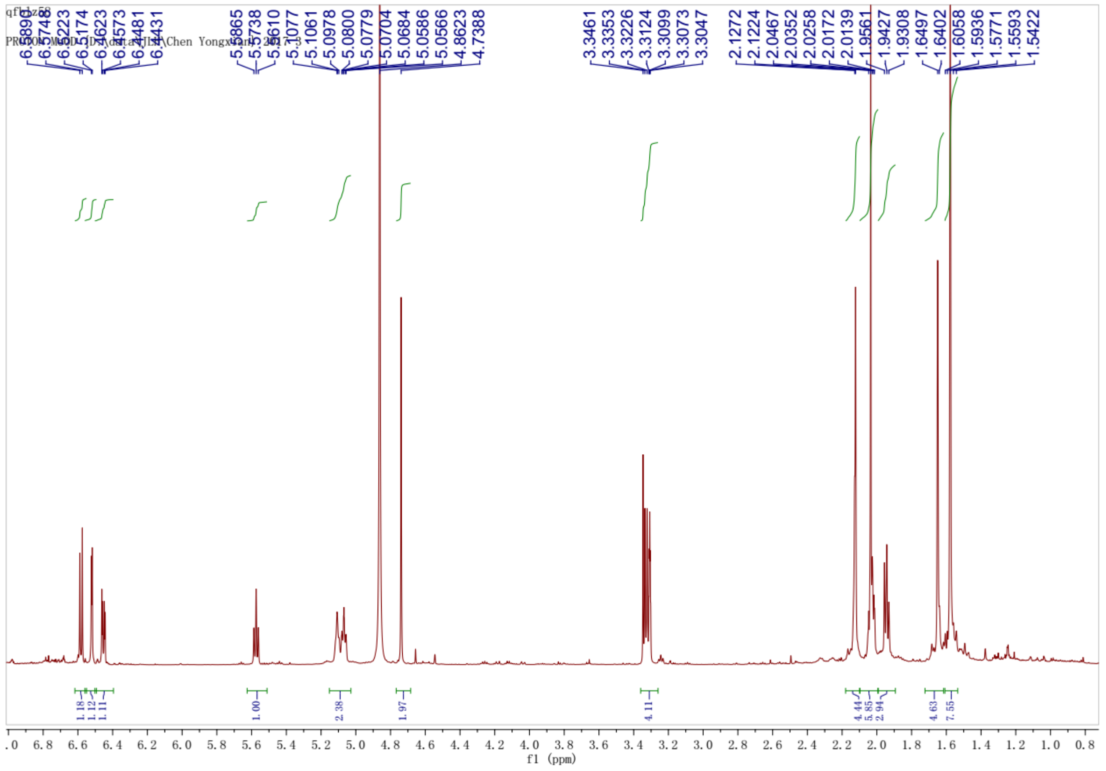


Figure S17. $^{1}$H NMR spectrum of **2** in methanol-$d_4$

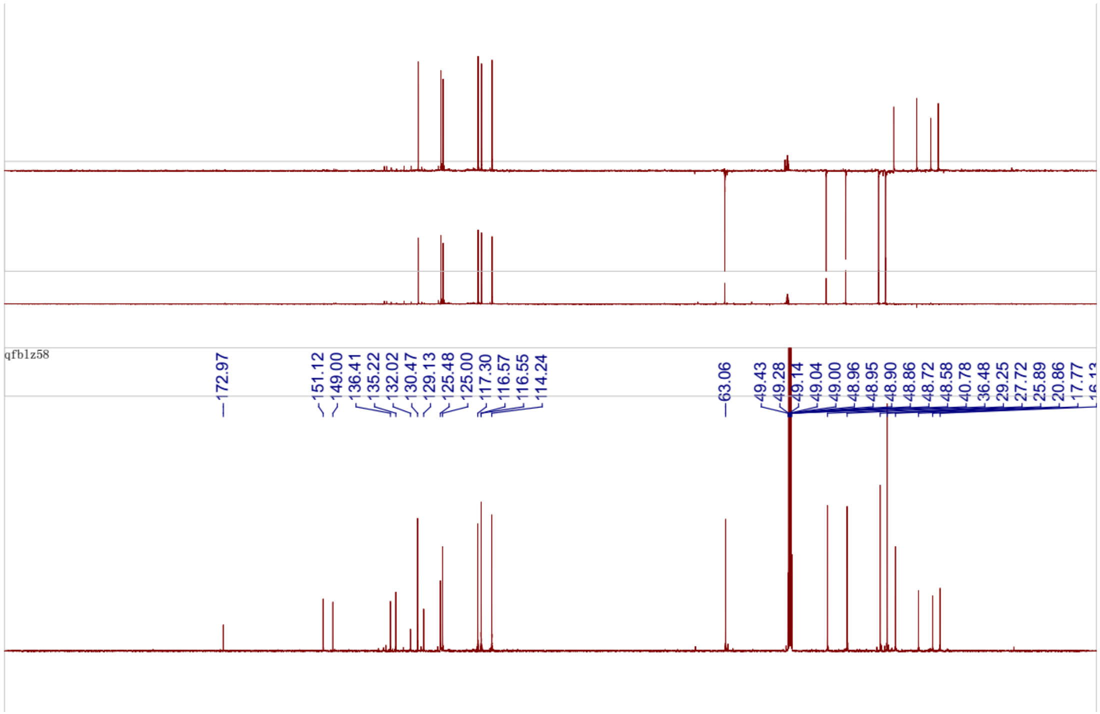


Figure S18. $^{13}$C NMR and DEPT spectra of **2** in methanol-$d_4$

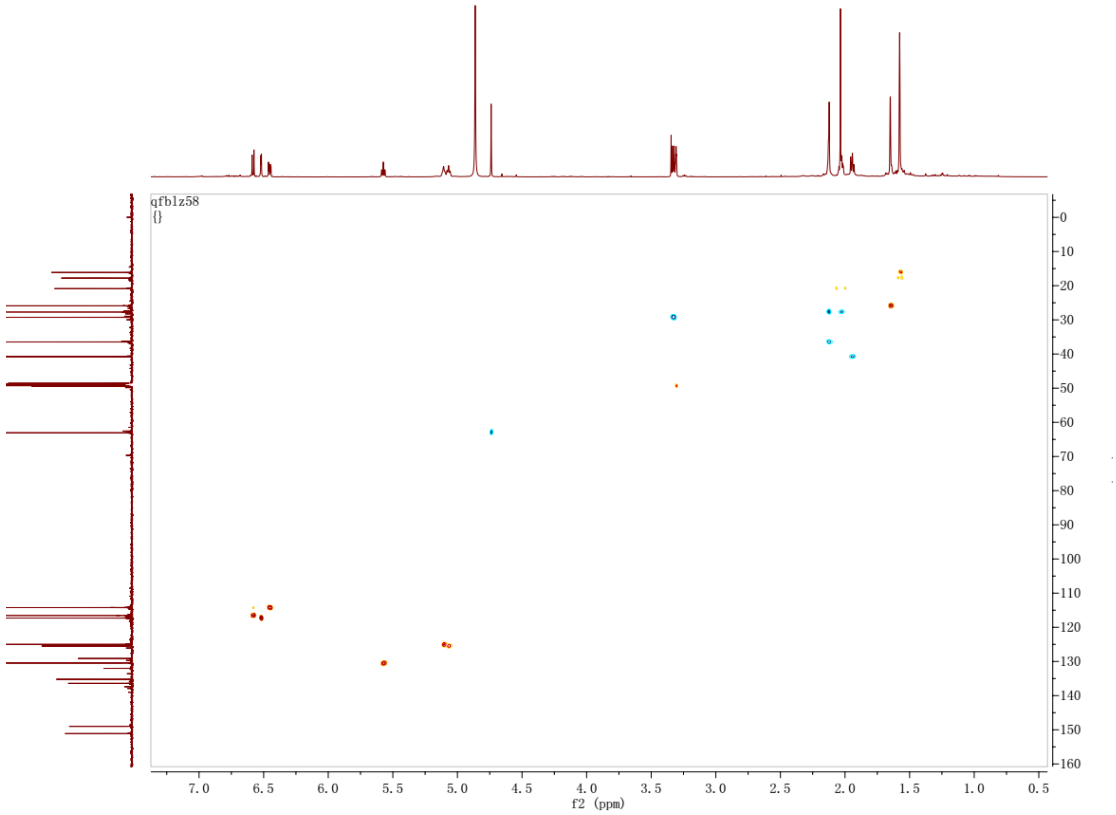


Figure S19. HSQC spectrum of **2** in methanol-$d_4$

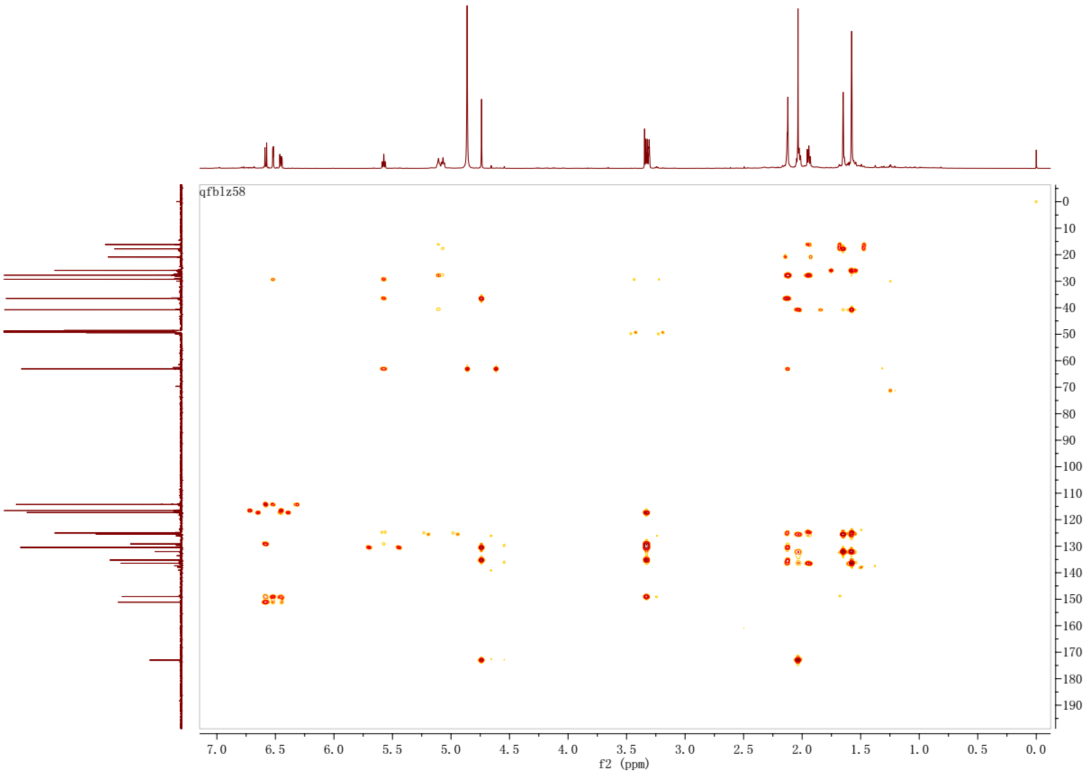


Figure S20. HMBC spectrum of **2** in methanol-$d_4$

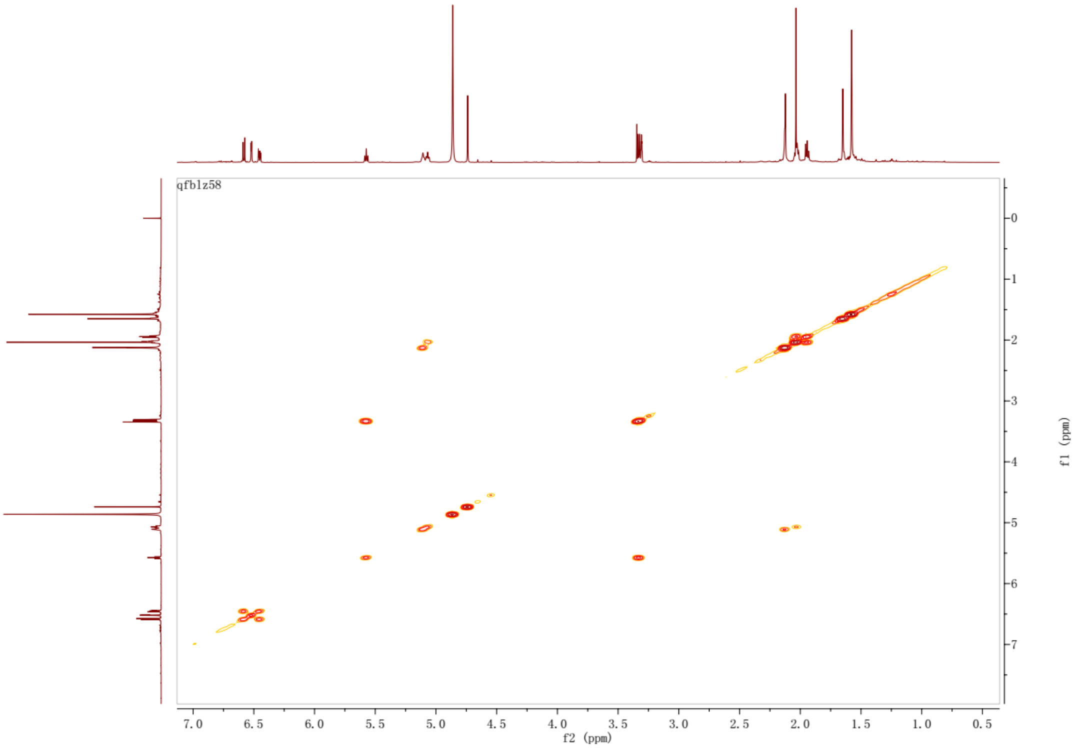


Figure S21. $^1$H-$^1$H COSY spectrum of **2** in methanol-$d_4$

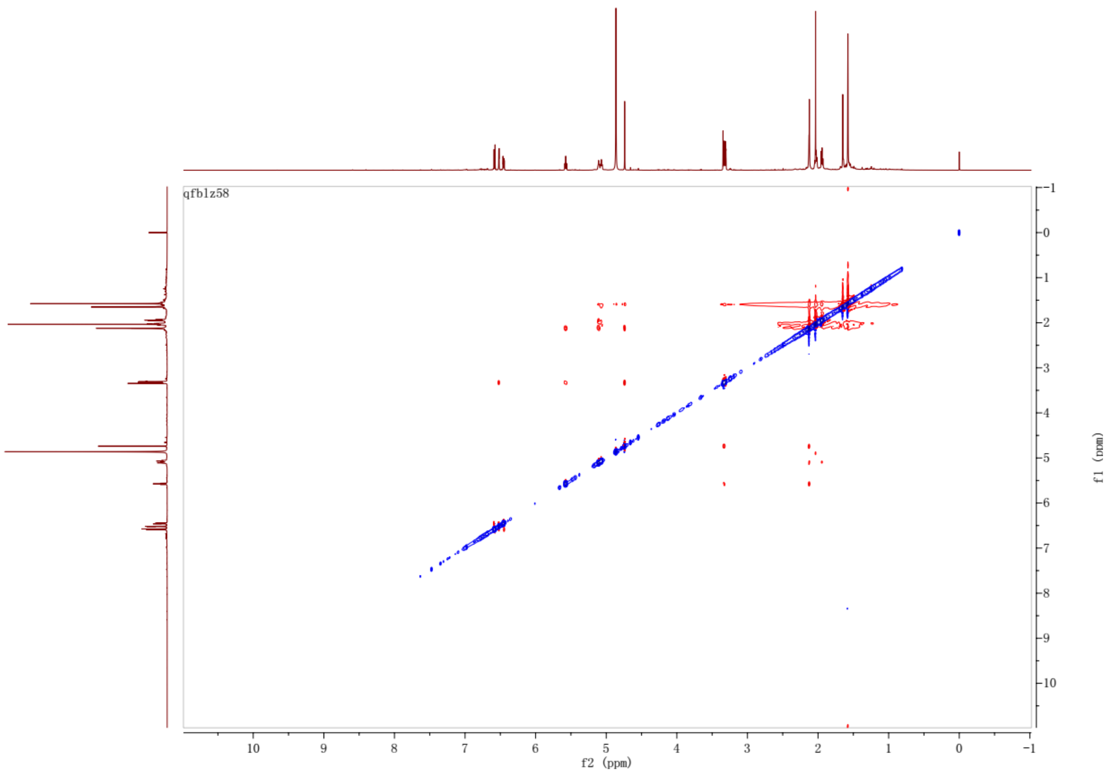


Figure S22. ROESY spectrum of **2** in methanol-$d_4$

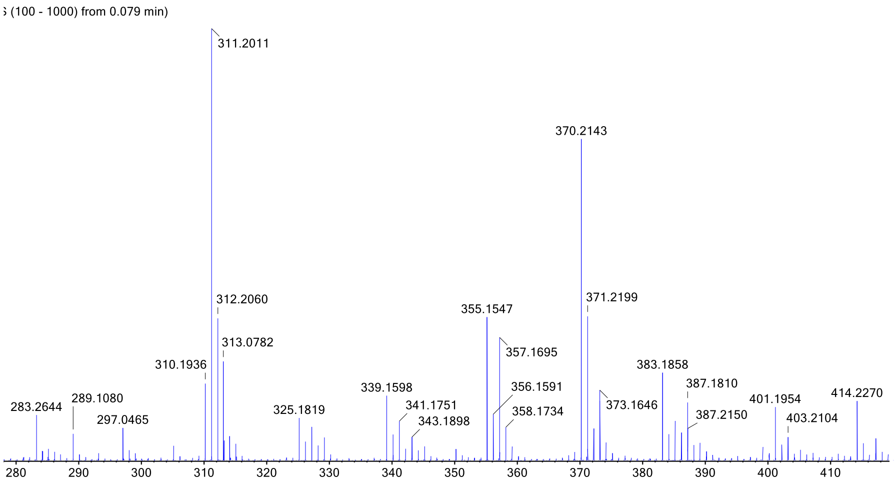


[M-H]$^-$ m/z 371.2199

| Hit | Formula | m/z | RDB | ppm |
|---|---|---|---|---|
| 1 | C23H31O4 | 371.2228 | 8.0 | -7.8 |

Figure S23. HRESIMS of **2**

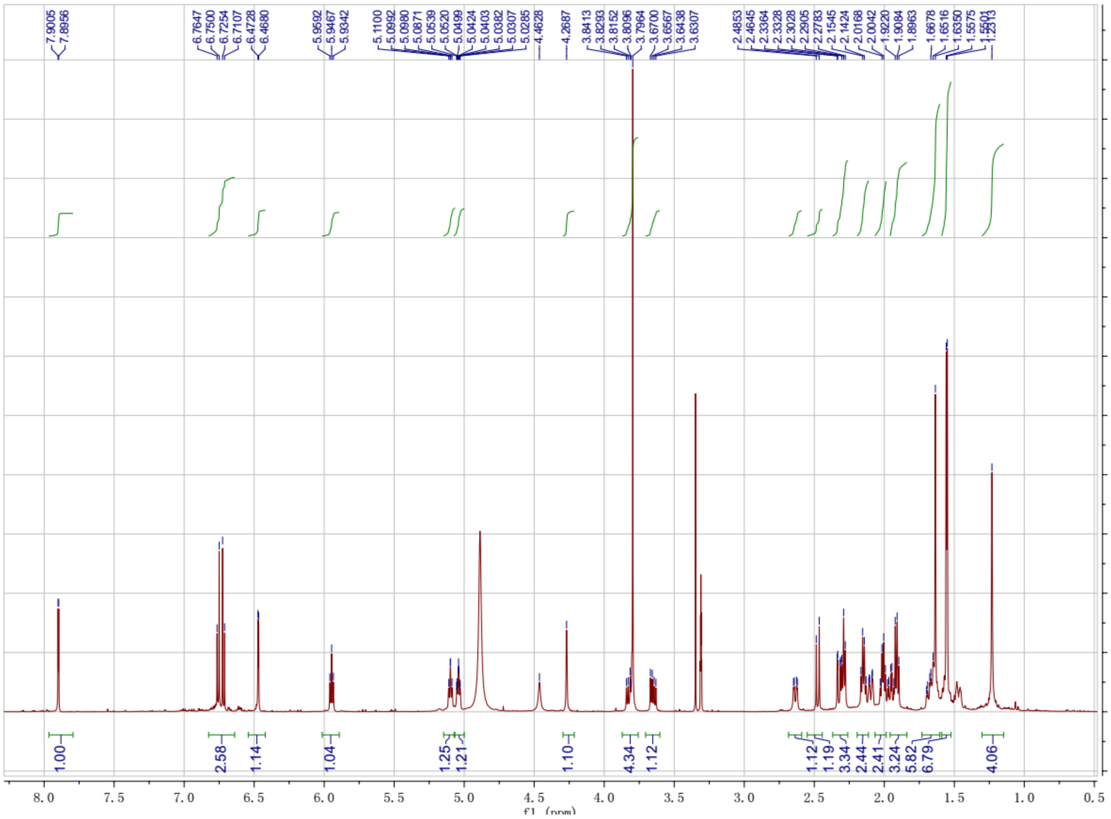

Figure S24. $^{1}$H NMR spectrum of **3** in methanol-$d_4$

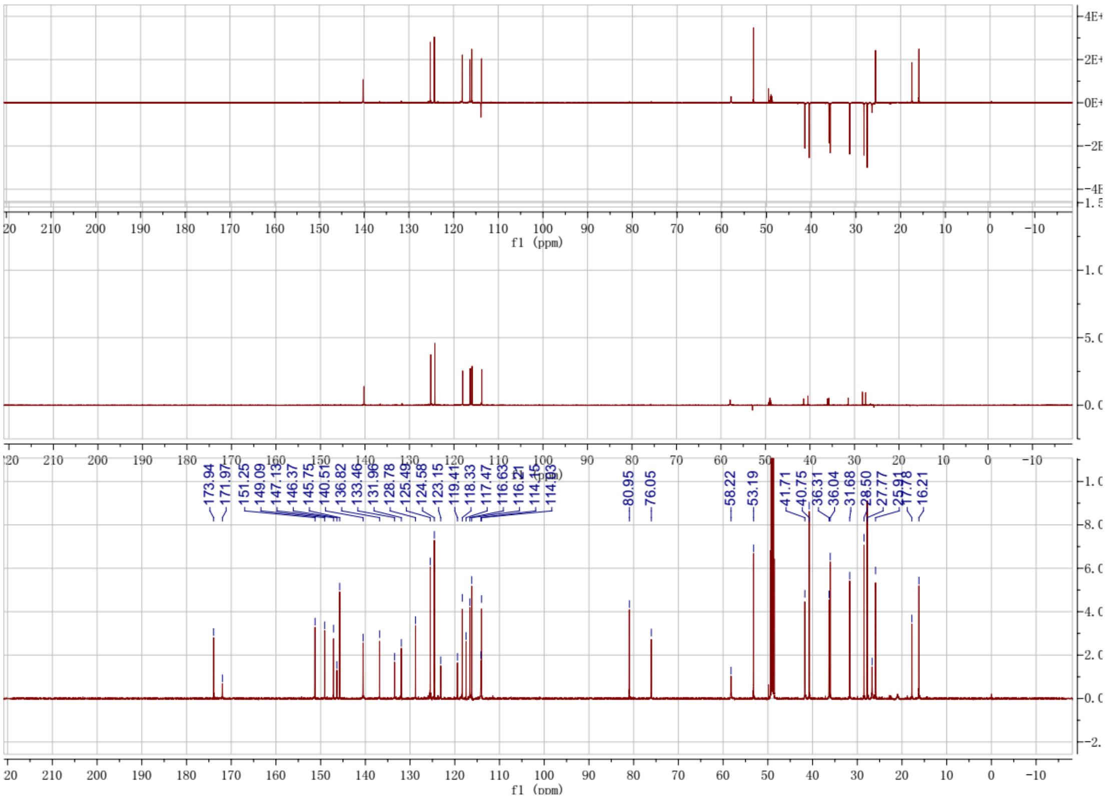

Figure S25. $^{13}C$ NMR and DEPT spectra **3** in methanol-$d_4$

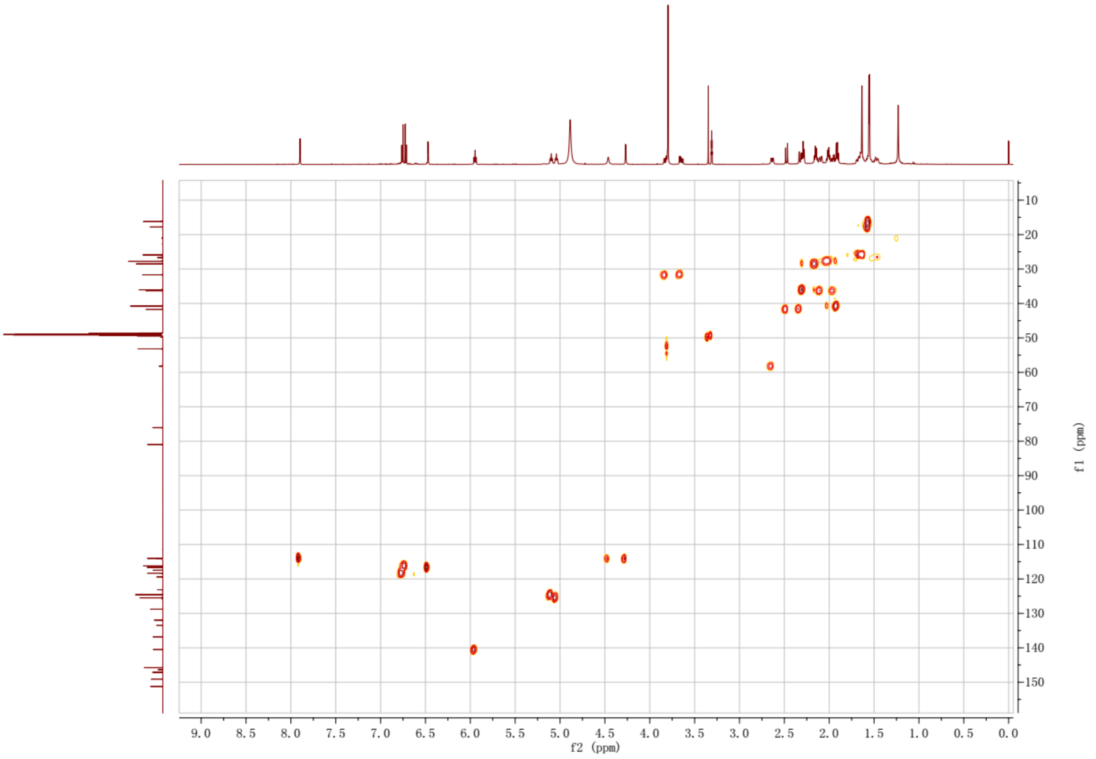

Figure S26. HSQC spectrum of **3** in methanol-$d_4$

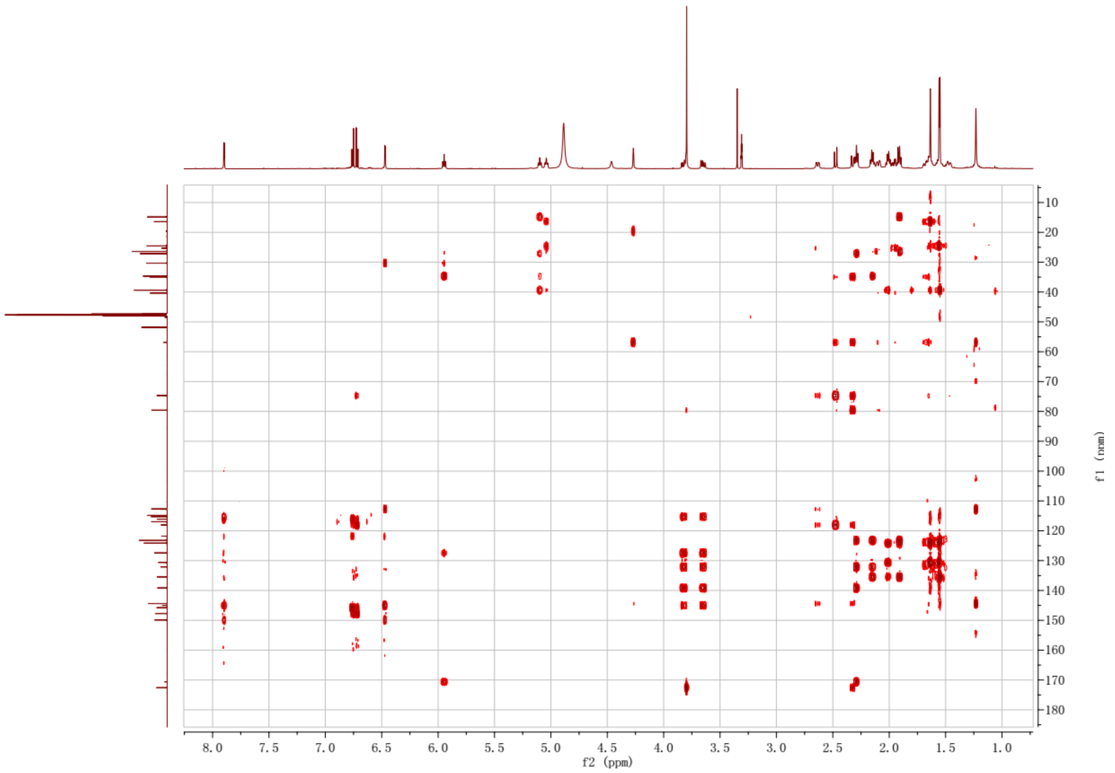


Figure S27. HMBC spectrum of **3** in methanol-$d_4$

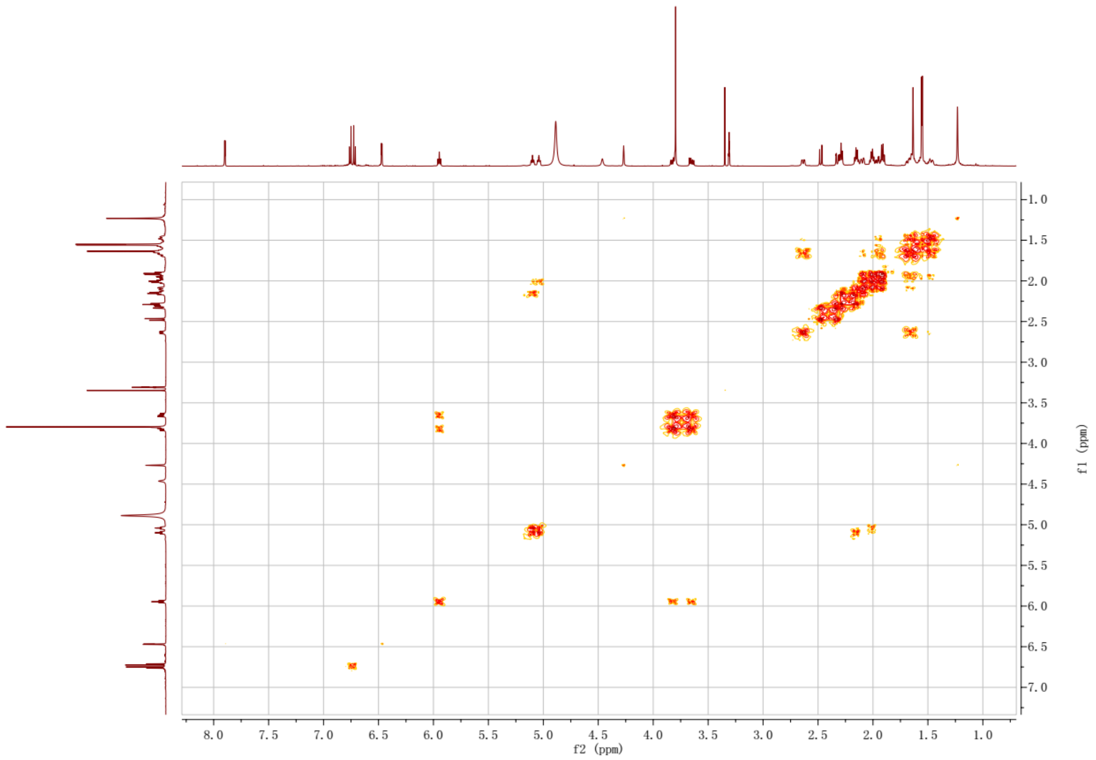


Figure S28. $^1$H-$^1$H COSY spectrum of **3** in Methanol-$d_4$

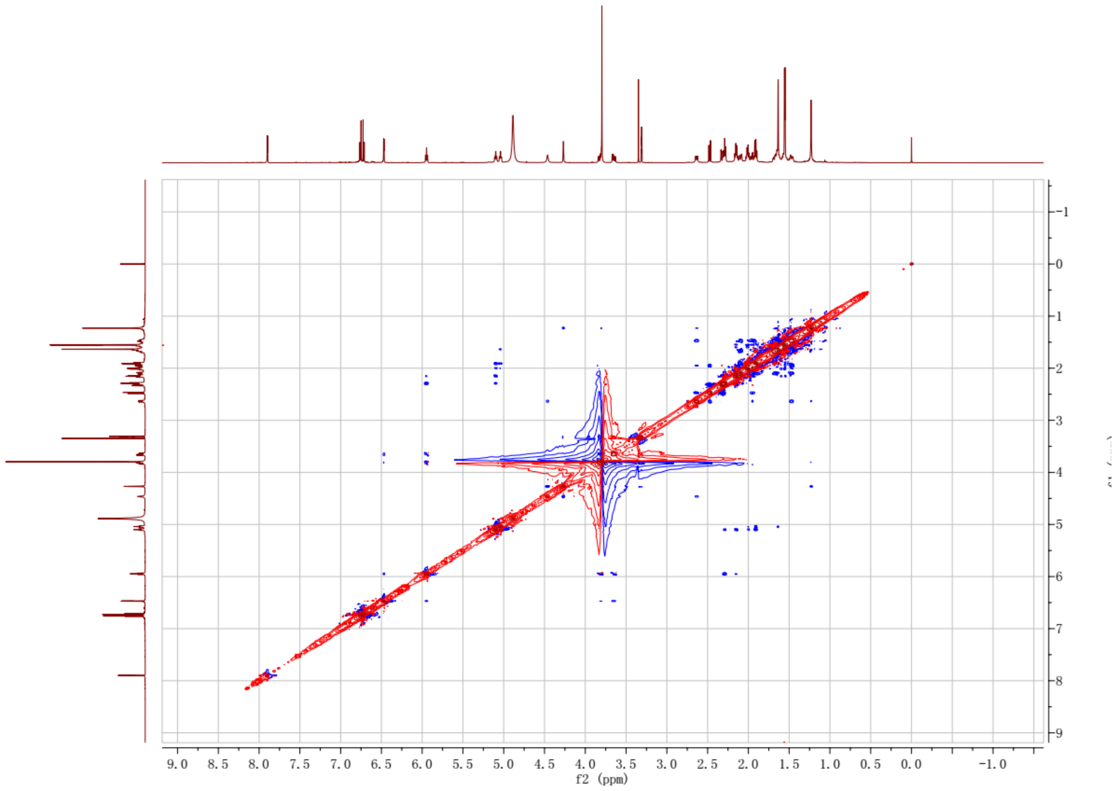


Figure S29. ROESY spectrum of **3** in methanol-$d_4$

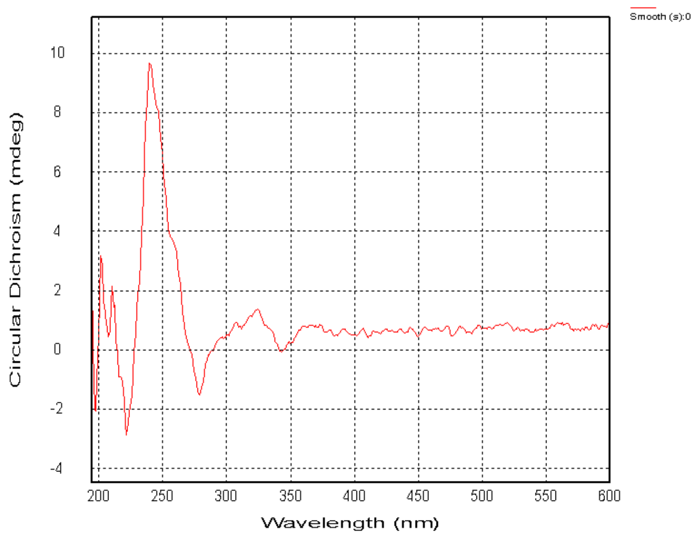


Figure S30 CD spectrum of (+)-**3**

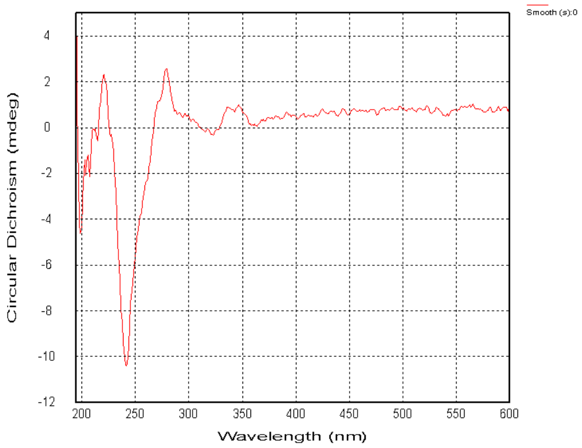


Figure S31 CD spectrum of (–)-**3**

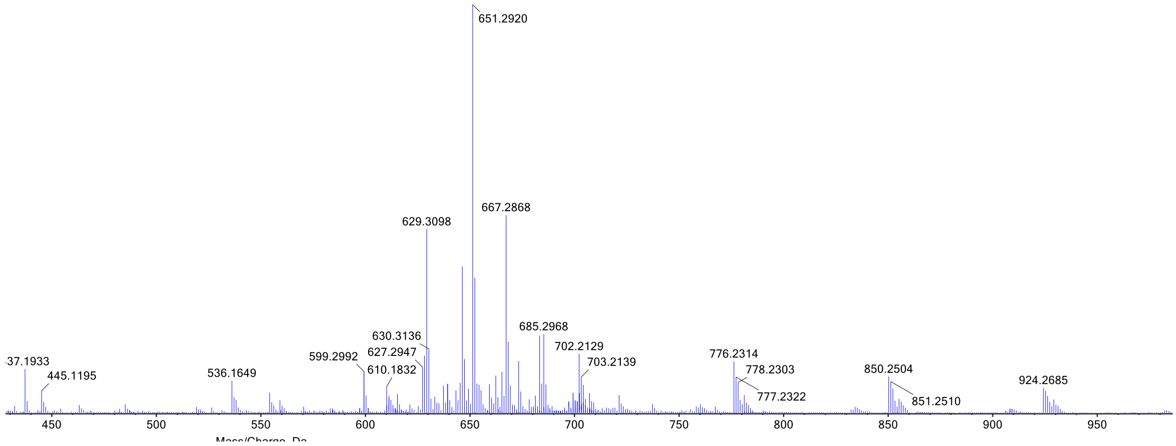


$[M+H]^+$ m/z 629.3098

| Hit | Formula | m/z | RDB | ppm |
|---|---|---|---|---|
| 1 | $C_{38}H_{45}O_8$ | 629.3109 | 17.0 | -1.7 |

Figure S32. HRESIMS of **3**

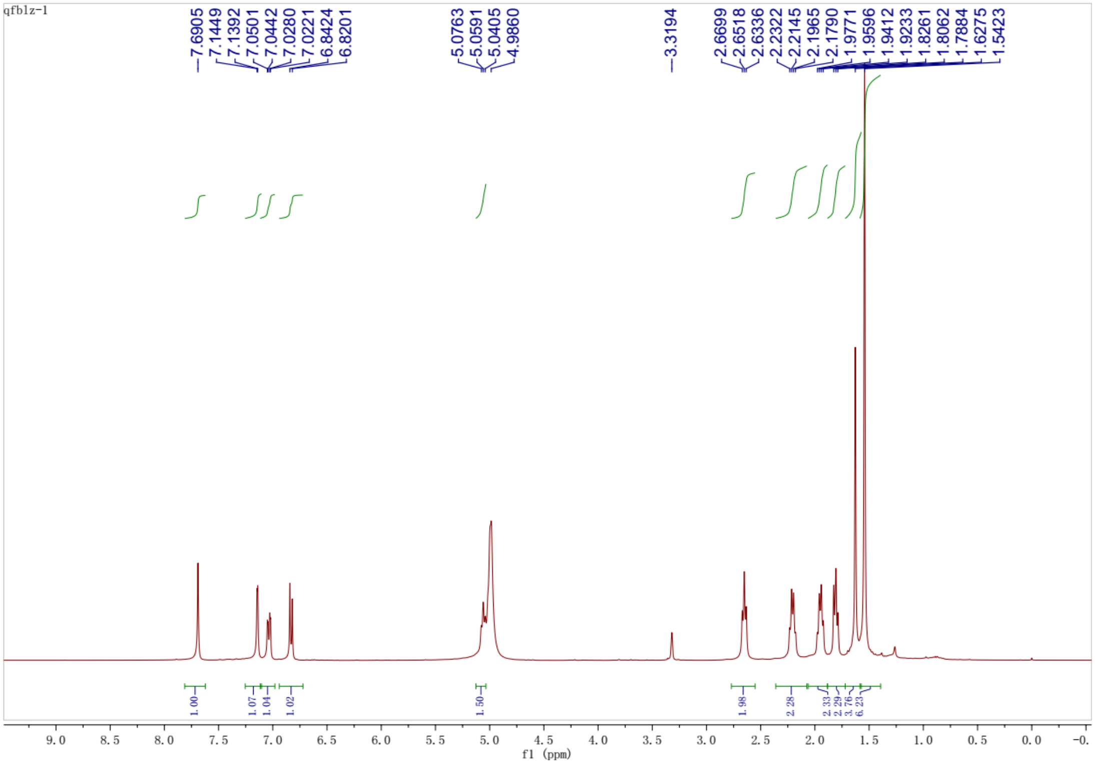

Figure S33.$^{1}$H NMR spectrum of **4** in methanol-$d_4$

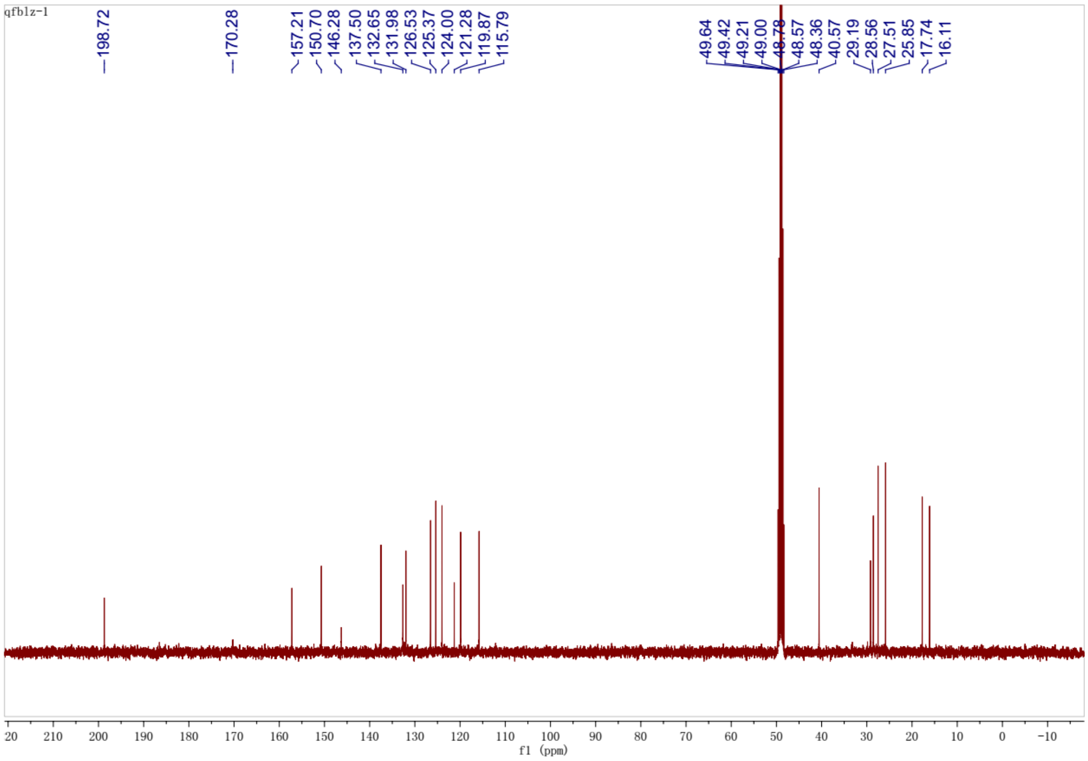

Figure S34.$^{13}$C NMR spectrum of **4** in methanol-$d_4$

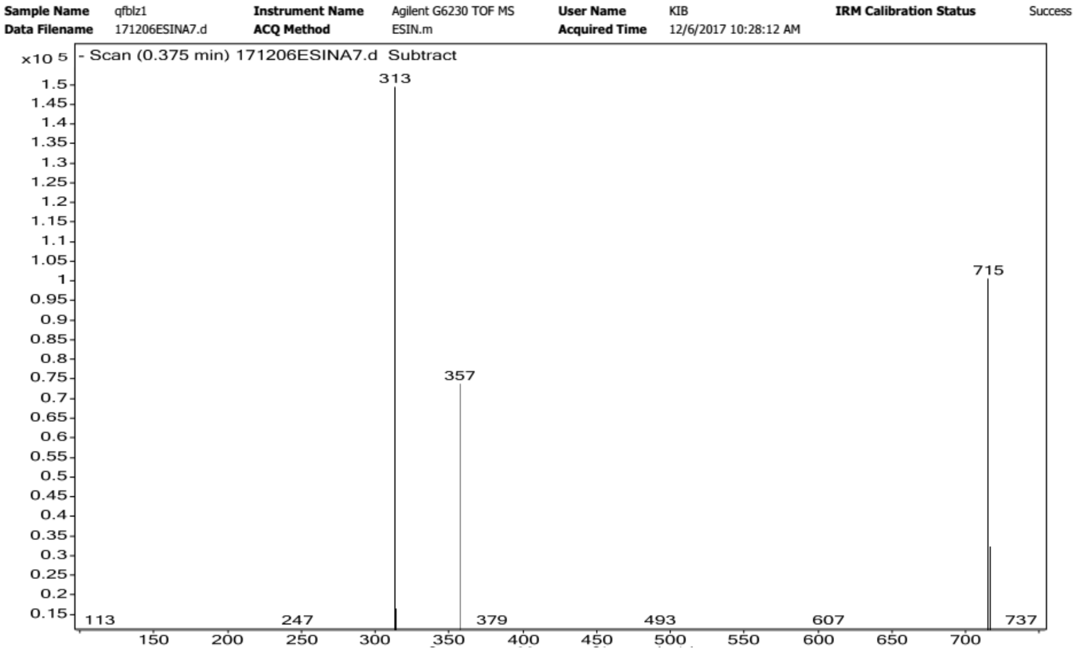


Figure S35. ESIMS of **4**

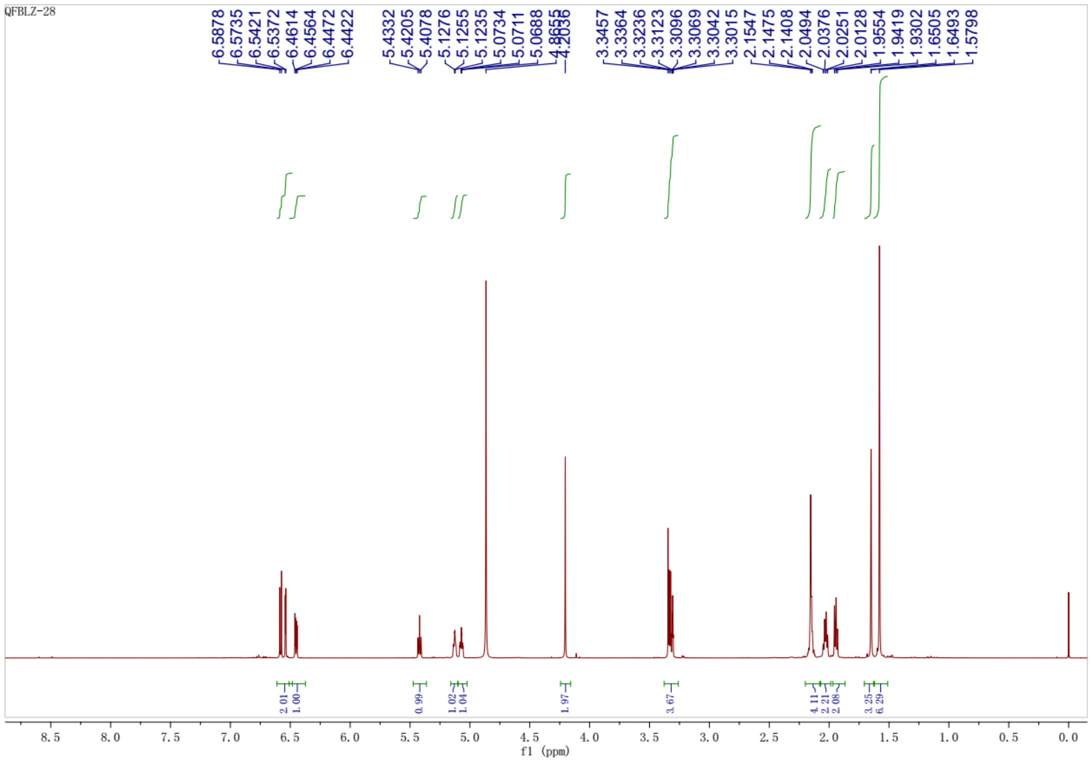


Figure S36.$^{1}$H NMR spectrum of **5** in methanol-$d_4$

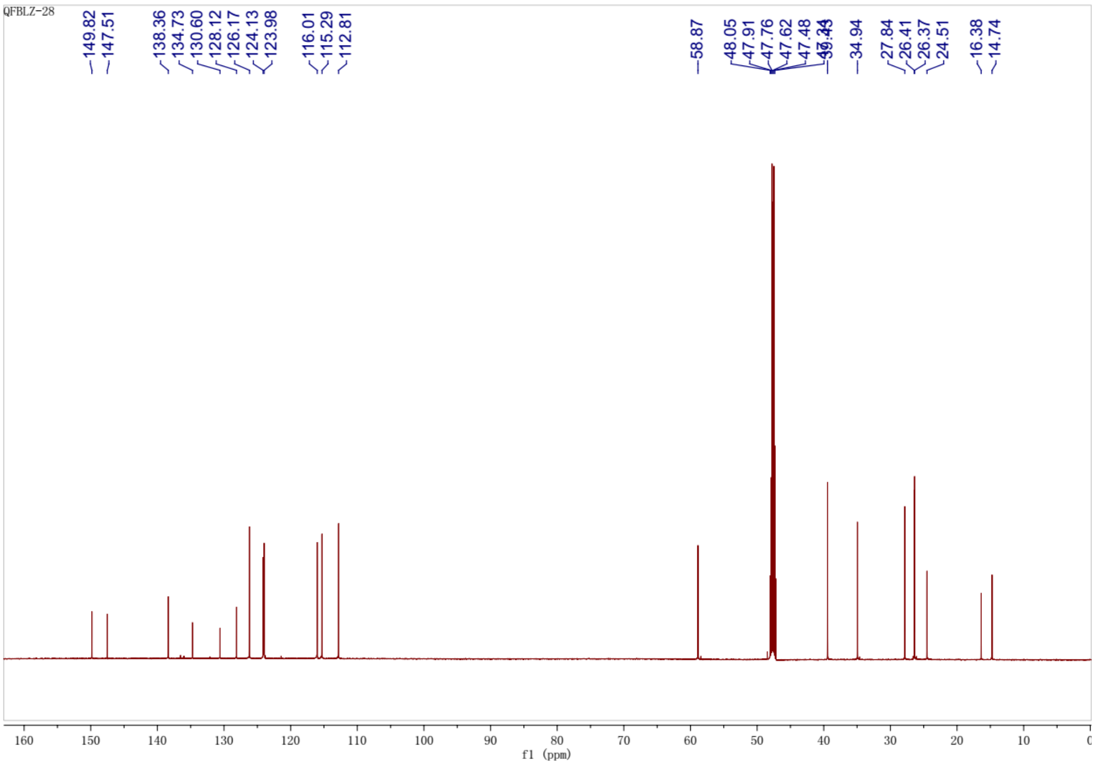


Figure S37.$^{13}$C NMR spectrum of **5** in methanol-$d_4$

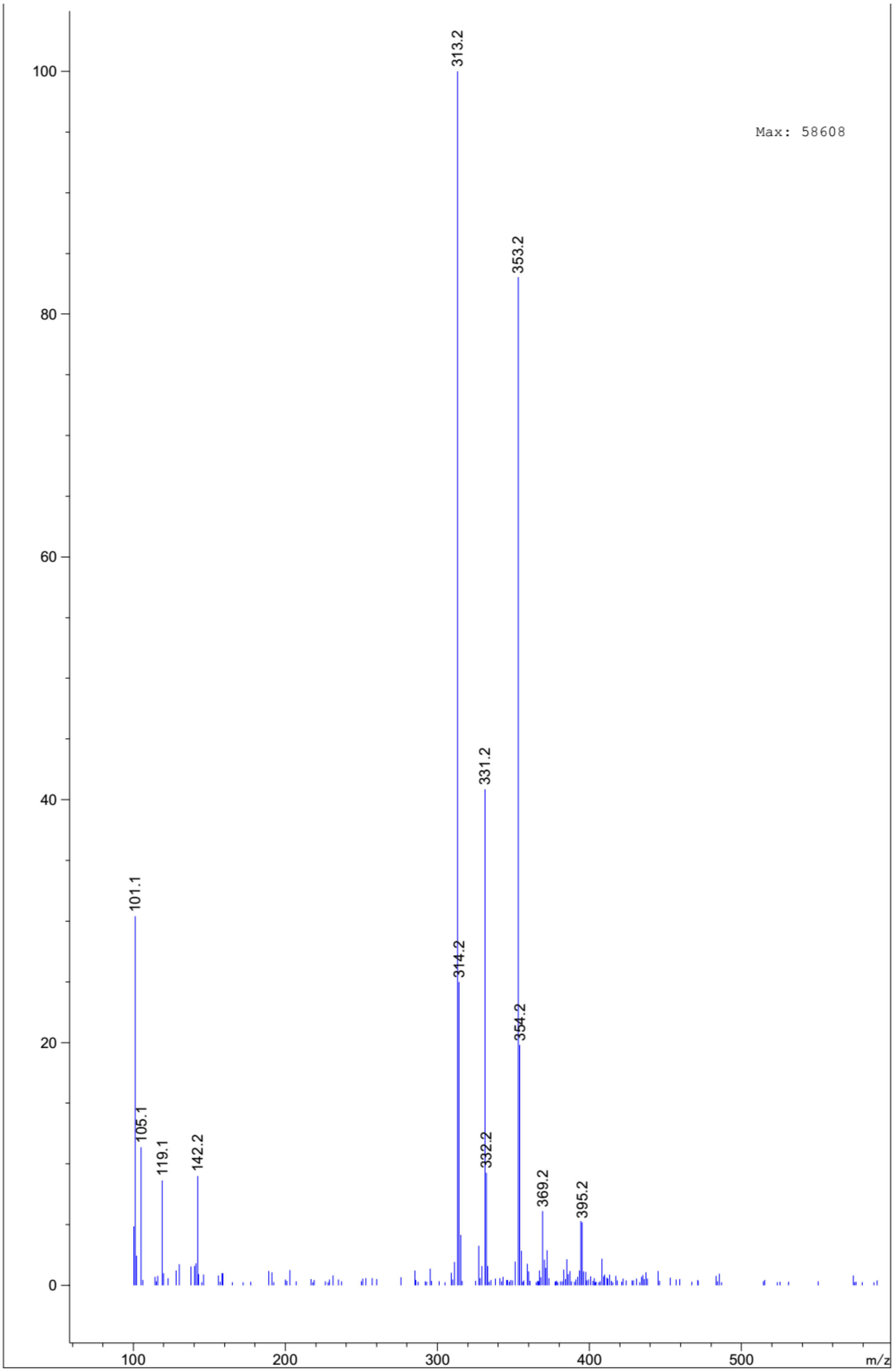


Figure S38. ESIMS of **5**

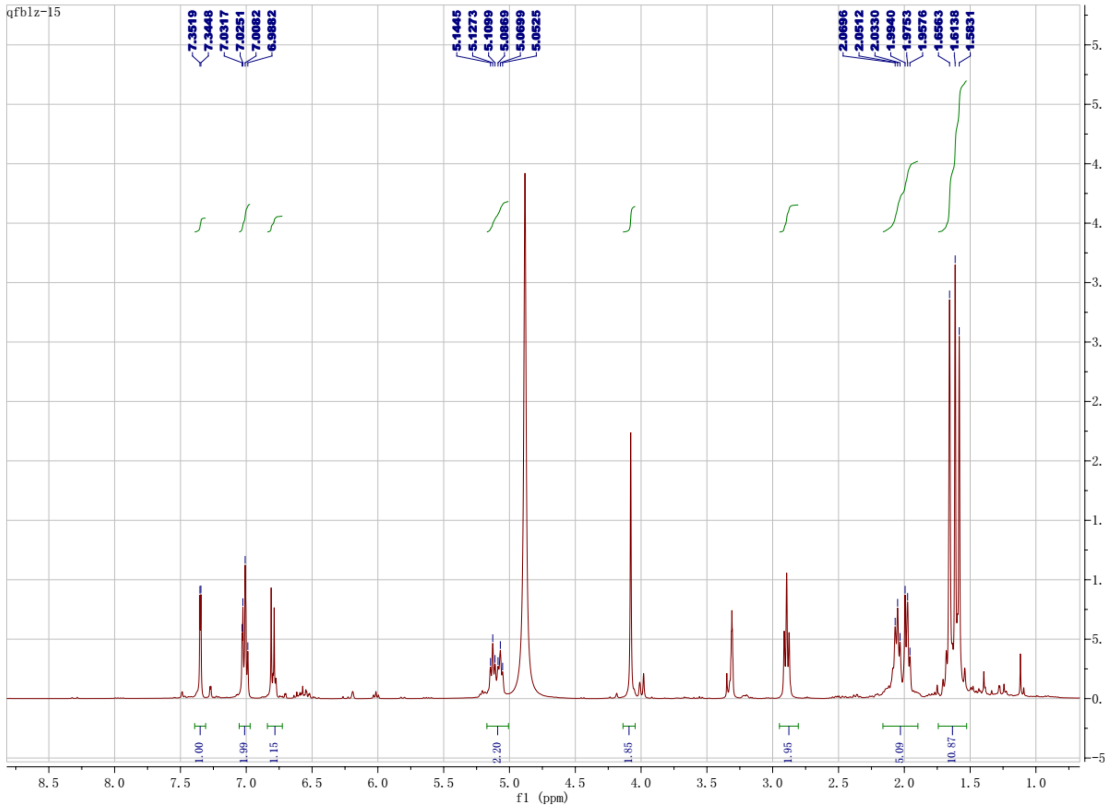


Figure S39.$^{1}$H NMR spectrum of **6** in methanol-$d_4$

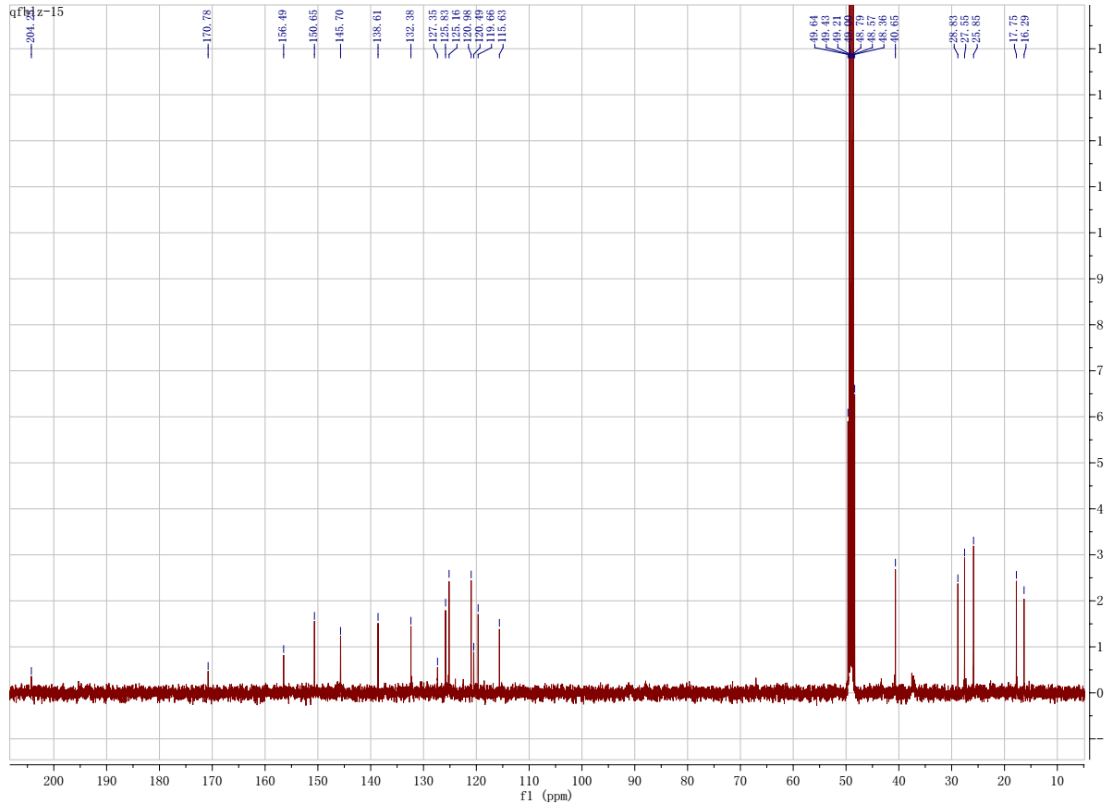


Figure S40.$^{13}$C NMR spectrum of **6** in methanol-$d_4$

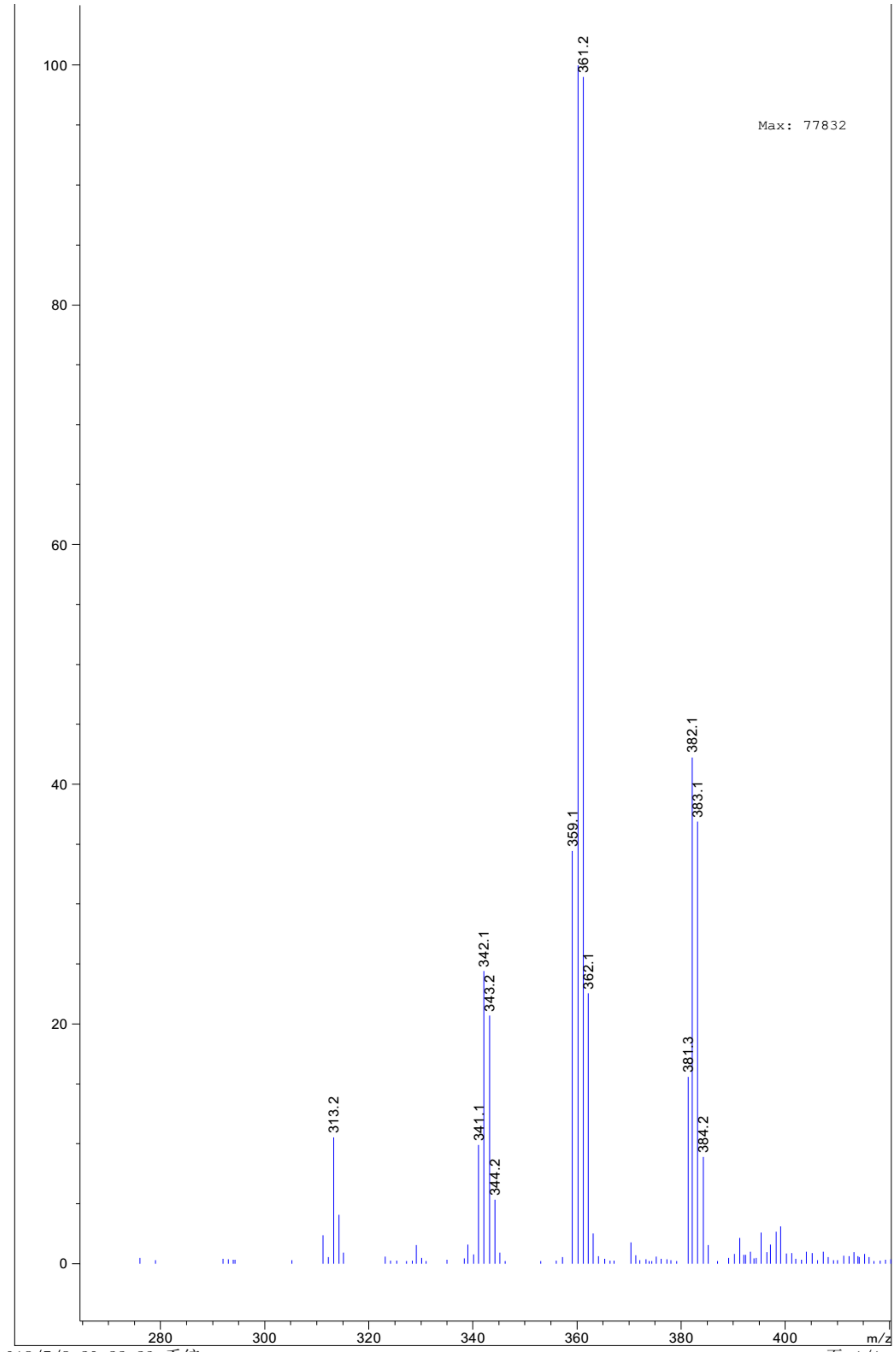


Figure S41. ESIMS of **6**

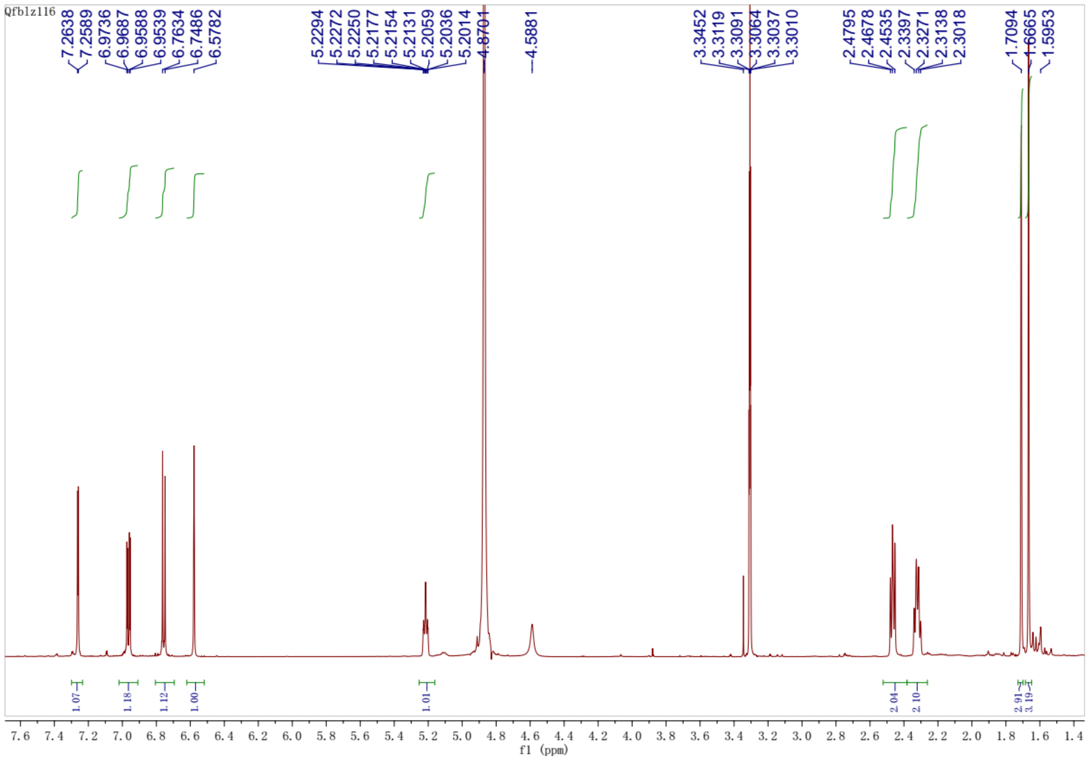


Figure S42.$^{1}$H NMR spectrum of **7** in methanol-$d_4$

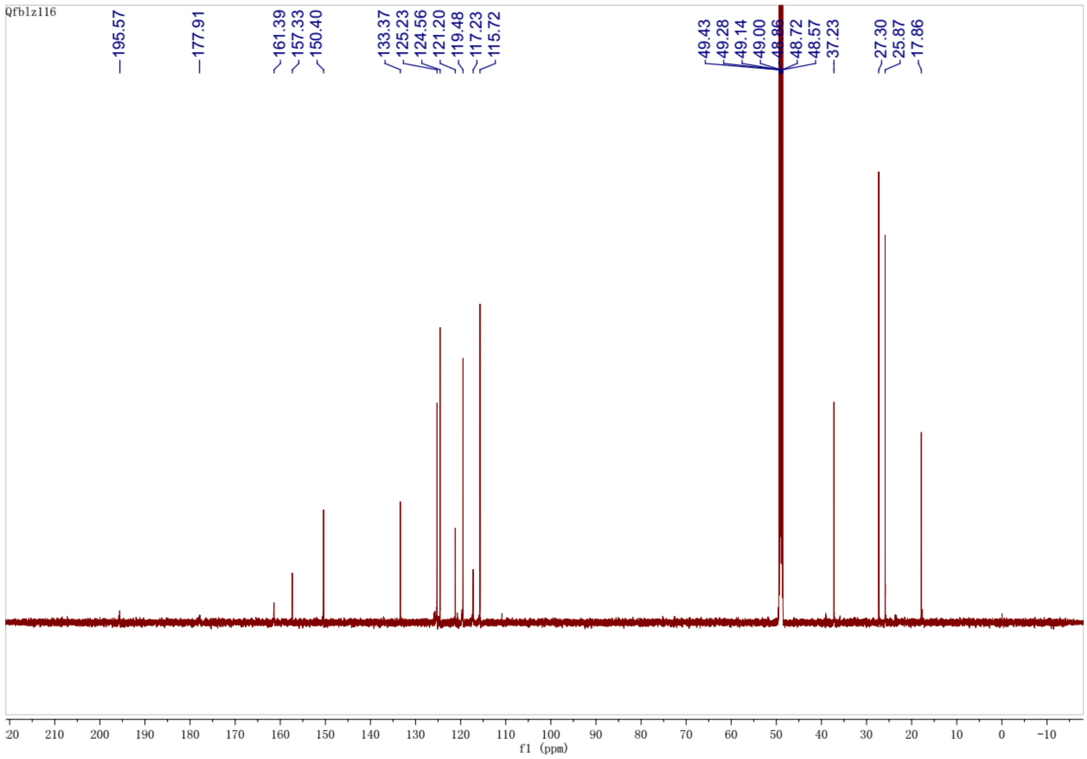


Figure S43.$^{13}$C NMR spectrum of **7** in methanol-$d_4$

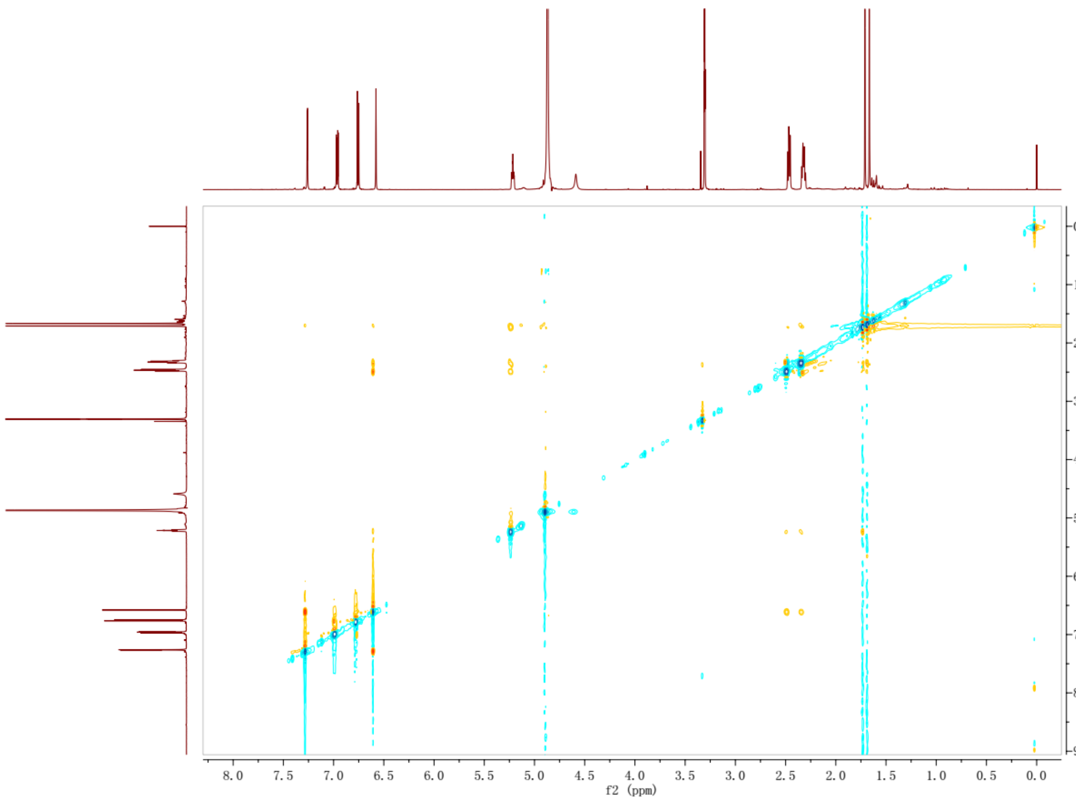


Figure S44. ROESY spectrum of **7** in methanol-$d_4$

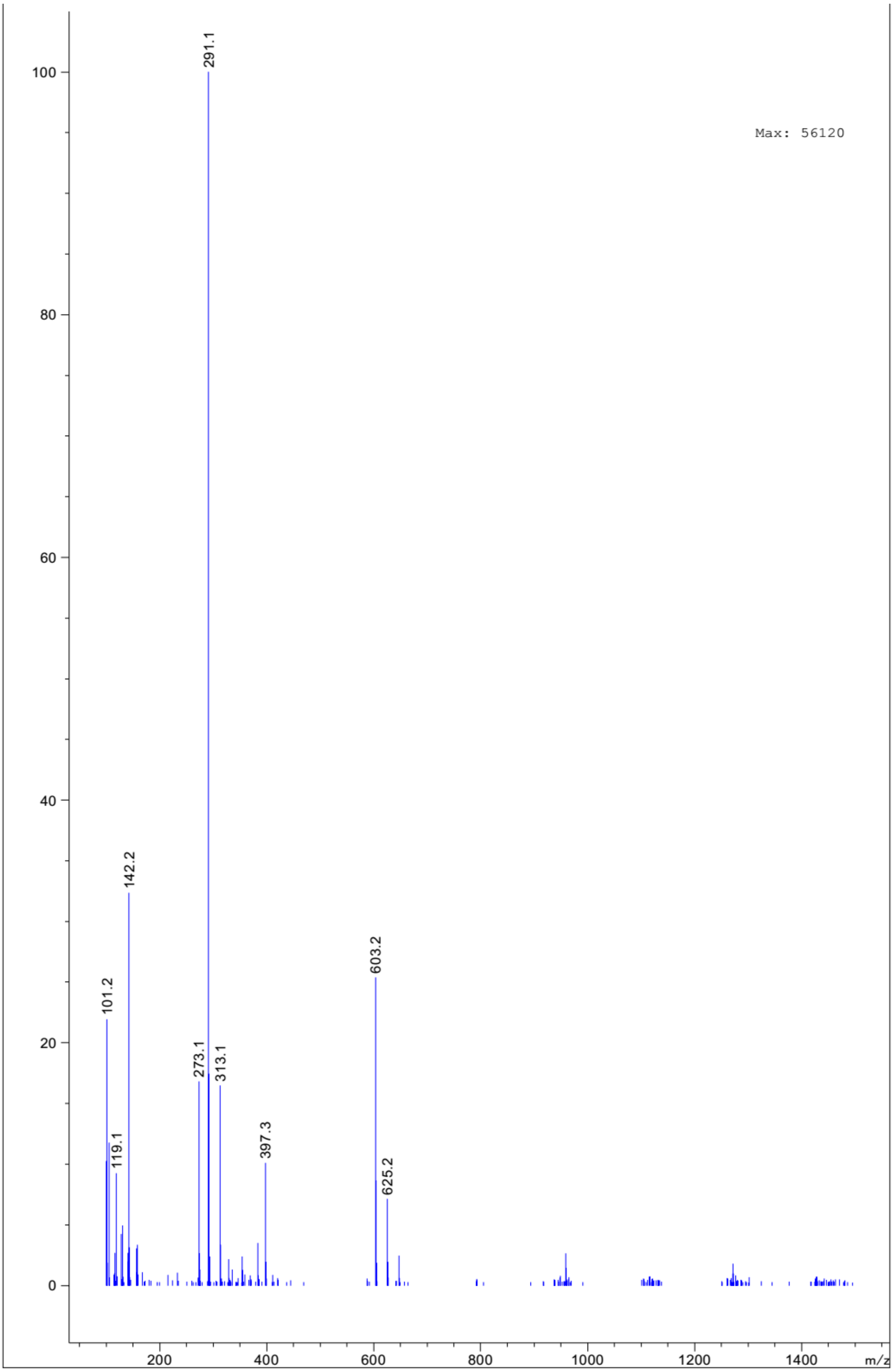


Figure S45. ESIMS of **7**

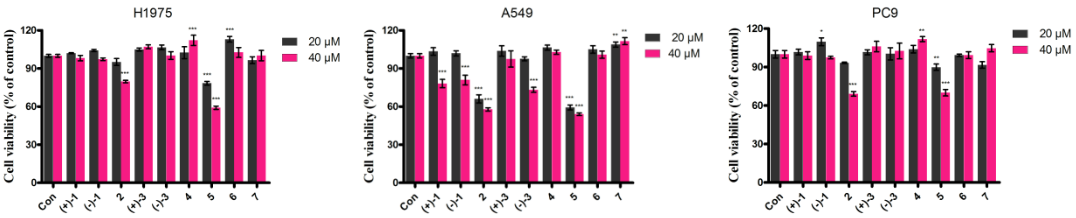


Figure S46. Cytotoxicity of the compounds toward three human lung cancer cell lines was measured using *TransDetect*® Cell Counting Kit
